# The Last Frontier: Catch Records of White Sharks (*Carcharodon carcharias*) in the Northwest Pacific Ocean

**DOI:** 10.1371/journal.pone.0094407

**Published:** 2014-04-16

**Authors:** Heather M. Christiansen, Victor Lin, Sho Tanaka, Anatoly Velikanov, Henry F. Mollet, Sabine P. Wintner, Sonja V. Fordham, Aaron T. Fisk, Nigel E. Hussey

**Affiliations:** 1 Great Lakes Institute for Environmental Research, University of Windsor, Windsor, Ontario, Canada; 2 Independent Researcher, Taipei, Taiwan; 3 School of Marine Science and Technology, Tokai University, Shimizu, Shizuoka, Japan; 4 Division of Marine and Freshwater Biological Resources, Sakhalin Research Institute of Fisheries & Oceanography, Yuzhno-Sakhalinsk, Russia; 5 Moss Landing Marine Laboratories, Moss Landing, California, United States of America; 6 Monterey Bay Aquarium, Monterey, California, United States of America; 7 KwaZulu-Natal Sharks Board, Umhlanga Rocks, South Africa; 8 Biomedical Resource Unit, University of KwaZulu-Natal, Durban, South Africa; 9 Shark Advocates International (a project of The Ocean Foundation), Washington, DC, United States of America; University of Sussex, United Kingdom

## Abstract

White sharks are highly migratory apex predators, globally distributed in temperate, sub-tropical, and tropical waters. Knowledge of white shark biology and ecology has increased recently based on research at known aggregation sites in the Indian, Atlantic, and Northeast Pacific Oceans; however, few data are available for the Northwest Pacific Ocean. This study provides a meta-analysis of 240 observations of white sharks from the Northwest Pacific Ocean between 1951 and 2012. Records comprise reports of bycatch in commercial fisheries, media accounts, personal communications, and documentation of shark-human interactions from Russia (n = 8), Republic of Korea (22), Japan (129), China (32), Taiwan (45), Philippines (1) and Vietnam (3). Observations occurred in all months, excluding October-January in the north (Russia and Republic of Korea) and July-August in the south (China, Taiwan, Philippines, and Vietnam). Population trend analysis indicated that the relative abundance of white sharks in the region has remained relatively stable, but parameterization of a 75% increase in observer effort found evidence of a minor decline since 2002. Reliably measured sharks ranged from 126–602 cm total length (TL) and 16–2530 kg total weight. The largest shark in this study (602 cm TL) represents the largest measured shark on record worldwide. For all countries combined the sex ratio was non-significantly biased towards females (1∶1.1; n = 113). Of 60 females examined, 11 were confirmed pregnant ranging from the beginning stages of pregnancy (egg cases) to near term (140 cm TL embryos). On average, 6.0±2.2 embryos were found per litter (maximum of 10) and gestation period was estimated to be 20 months. These observations confirm that white sharks are present in the Northwest Pacific Ocean year-round. While acknowledging the difficulties of studying little known populations of a naturally low abundance species, these results highlight the need for dedicated research to inform regional conservation and management planning.

## Introduction

Details on the population status and spatial/temporal distribution of threatened species are critical to focus conservation efforts [1], [2]. Typically, species that have globally distributed populations are exposed to a range of region-specific threats and pressures and consequently management actions require data for each region [3]–[5]. Regional declines of large marine predators including elasmobranchs have been documented [6], [7], with potential cascading effects on marine food webs [8]. For many large elasmobranch species, data on spatial and temporal distributions are limited due to their migratory behavior, the relative rarity of sightings, and the nature of the environment they inhabit [9], [10].

The white shark (*Carcharodon carcharias*) is a large (maximum size 6 m, [11], [12]) marine apex predator with a global distribution, occurring in temperate, sub-tropical, and tropical waters [13]–[16]. The life history characteristics of white sharks (e.g., natural low abundance, slow growth, late maturity, low fecundity) make them vulnerable to exploitation [17], [18]. Population estimates conducted in California [19], South Africa [20], [21], and Australia [22] indicate relatively low regional population sizes with genetic diversity constricted by philopatric behavior [23]. White sharks are classified as threatened (globally Vulnerable) by the International Union for Conservation of Nature (IUCN), listed on the Convention on International Trade of Endangered Species of Wild Fauna and Flora (CITES) Appendix II, listed on both Appendix I and II of the Convention on Migratory Species (CMS), and are protected by national legislation in Australia, Canada (Atlantic Ocean), Croatia, European Union, Maldives, Malta, Mexico, Namibia, New Zealand, South Africa, and in all United States waters (except in the Western Pacific) [24], [25]. For the countries of the Northwest Pacific Ocean, participation in CITES and CMS is variable, while none appear to impose complete national white shark protection. Specifically, the only domestic, white shark-specific conservation measure identified in the region applies in China, but permits with unclear conditions may allow for some take (Table 1).

**Table 1:** Participation in International Agreements for Countries in the Northwest Pacific Ocean.

Fishing Entity	CMS Party	CMS Shark MoU Signatory	CITES Party	Finning Ban	WCPFC* Member	IOTC* Member
**Russia**	No	No	Yes	No	No	No
**China**	No	No	Yes	*	Yes	Yes
**Japan**	No	No	Yes, Reservation on white shark listing	Yes, with weak standards & some exceptions	Yes	Yes
**Philippines**	Yes	Yes	Yes	*	Yes	Yes
**Vietnam**	No	No	Yes	No	No	No
**Republic of Korea**	No	No	Yes	No	Yes	Yes
**Taiwan**	No	No	No	Yes, used fin to carcass ratio; now phasing in fins attached rule	Yes	Cannot become member because of lack of membership in UN bodies

*These RFMOs have adopted finning bans (based on a 5% fin to carcass ratio limit). Members are obligated to adopt domestic regulations in line with RFMO measures.

**Figure 1:**
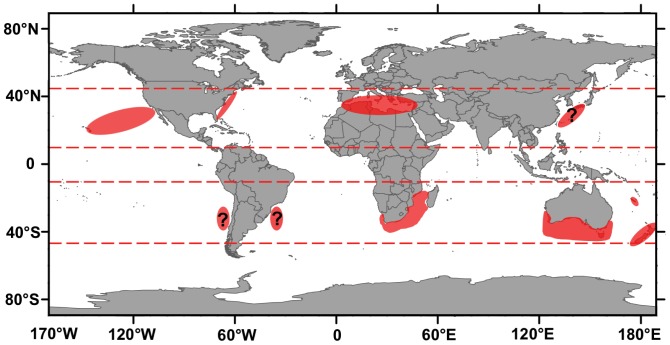
Global hotspots of white sharks. Known global white shark aggregation sites. Question marks indicate suitable latitudinal ranges where white sharks may occur, but little is known about current population trends.

The white shark is a charismatic species and consequently one of the most studied and protected shark species [26]; however, there is still much to learn about their basic biology and ecology. The majority of recent research on white shark movement and feeding ecology has focused on regional population hotspots or aggregation sites in the Northeast Pacific, South Africa, and surrounding Australia (including New Zealand and New Caledonia), with only a few studies examining the lesser-known populations of the Mediterranean [27]–[29] and the Northwest Atlantic [30] (Fig. 1). For the Northwest Pacific region, there is a paucity of available data on white sharks with only a few reports of incidental captures and white shark-human interactions (bites) from Japan [31]–[36] and Russia [37], [38]. This disparity in research focus is so large that the Proceedings book of the last international white shark symposium [39] contained no data for the Northwest Pacific Ocean [26]. Currently, the frequency of occurrence and geographical extent of the population of white sharks in this region is unknown. Based on repeated long distance migrations recorded in South Africa and the Northeast Pacific [13], [16], [40], [41], it is possible that white sharks transit to the Northwest Pacific from other regional populations, such as Australia, however, evidence suggests that white sharks caught off Japan form a genetically distinct population that has a vastly different growth rate from the Northeast Pacific and South African populations [36]. It is therefore likely that there is a separate resident population of white sharks inhabiting the Northwest Pacific region.

Considering the conservation concern for white sharks, the lack of protective measures in the Northwest Pacific region and the limited data available for the associated population, the aims of this study were to; i) provide a comprehensive record of the distribution of white shark catches and sightings in the Northwest Pacific Ocean, ii) describe spatio-temporal patterns in white shark occurrence, iii) examine relative population trends over the past 60 years, iv) document size and sex based trends, and v) provide details on the reproductive biology of females. These data provide a baseline to stimulate further research and to inform regional management for this little known population.

## Methods

The study region of the Northwest Pacific Ocean was defined as the waters surrounding Russia, the Republic of Korea, Japan, China, Taiwan, the Philippines and Vietnam. A literature search was conducted using the ISI Web of Science. Search terms included: “white shark”, “*Carcharodon carcharias*” and “Russia or Republic of Korea or Japan or China or Taiwan or Philippines or Vietnam”. This resulted in 12 studies, six of which contained no relevant data for the Northwest Pacific Ocean and were therefore excluded. Regional observations of white sharks were defined as any records of fishery landings/discards, interactions with humans (bites), or museum specimens. To document white shark observations from media reports and websites, a search was conducted using Google. Personal communications and observations by the authors were also included. In Japan, the Ibaraki Prefectural Oarai Aquarium works directly with local fishermen, including those involved in set net fisheries, to document white shark catches.

### Data Analysis

Data were filtered to remove duplicate observations. The amount of information recorded for each white shark observation ranged from basic data that included only the country of capture to detailed records that included date of capture, city landed, method of capture, size (cm total length - TL), weight (kg), sex, and if the individual was pregnant. In most instances, the locations of white sharks were based on landing sites rather than actual catch locations (see Table S1). To examine population trends over time, a generalized linear model was fit to the observation data period to examine the magnitude of change in population relative to a set of reference dates following the approach developed by McPherson and Myers [42]. Values equal to one or less suggest a stable or increasing population, while values larger than one are indicative of a decline in relative abundance. This model enables testing of sensitivity to changes in observer effort over the reference period of 1951–2011. Consequently, +75%, 0% and −75% changes in observer effort were selected to incorporate a wide range of potential observer variation.

We documented whether length and weight were measured by scientifically trained staff or estimated by laypeople/untrained fishermen, from photos, or by jaw size. When both an estimate from a fishermen and calculated TL from jaws or photos were available, the TL from the latter was used. When multiple weight estimates were available the lowest estimate was retained. The length-weight relationship was calculated using the equation logW = log*a*+b*log TL where W is weight (kg), TL is total length (cm) and *a* and *b* are constants (where log *a* is the intercept and *b* is the slope). Significant differences by sex were tested using an analysis of covariance (ANCOVA) on log-transformed data. Since estimated weights were used for several individuals (Table S1), the validity of the data was tested using an ANCOVA with length-weight data obtained from white sharks caught in beach protection nets off KwaZulu-Natal, South Africa (KwaZulu-Natal Sharks Board, unpublished data), as well as global length-weight data collected by trained scientists [17], [27], [43] (see references therein). Pregnant females were excluded from this analysis.

Due to the number of observations where sex data were available (113, 47%), the sex ratio for each country was determined, but further statistical analysis, e.g. segregation by size and sex or temporal differences by sex, were not conducted. Embryos of pregnant individuals were categorized by size as early term (<40 cm TL), mid-term (between 40 and 100 cm TL) or full term (>100 cm TL) [17], [35], [44]. For individuals where embryo size was described by stage, this description was maintained. To estimate gestation length, data from embryos and free-swimming juveniles from the Northern and Southern Hemispheres (Table S2) were combined on one time scale, where January equals month 0 in the Northern Hemisphere and July equals month 0 in the Southern Hemisphere. Linear regression was performed on the embryo size versus month of capture data and gestation length was determined using the slope of the regression, and the size at birth (we used 126 cm TL, the smallest free-swimming individual in the Northwest Pacific).

## Results

**Figure 2:**
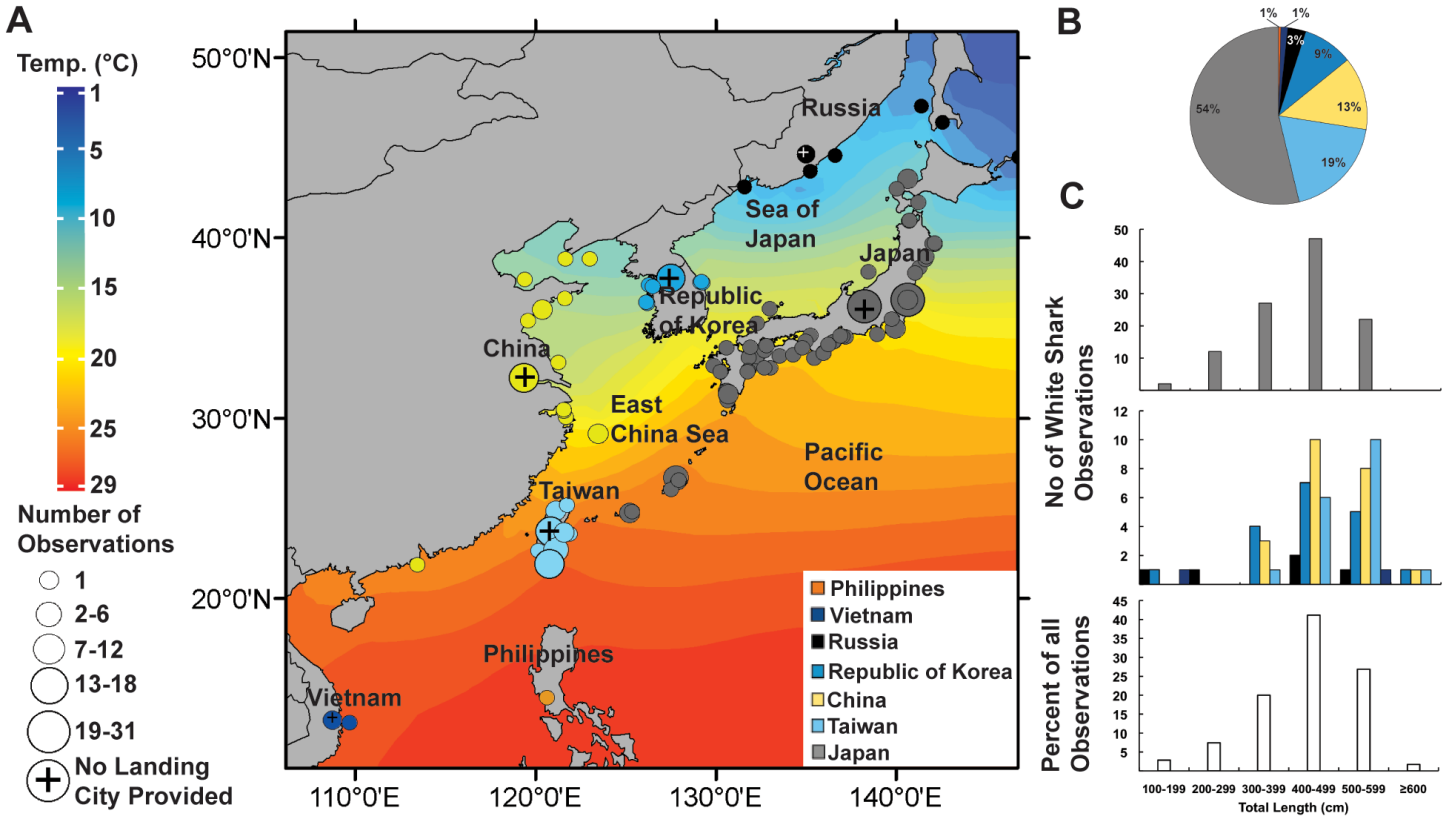
Geographical extent and size distribution of white shark observations. Color legend for country of observation occurrence applies to entire figure. A) Approximate location of observation or landing for individual white sharks. Circles on land indicate observations that only reported country of landing. Annual average sea surface temperature is indicated by color gradient. B) Percentage of white shark observations by country landed. C) Size of white shark observed by country landed (top two panels) and as a percent of all observations (bottom panel). Note–the Philippines observations did not have an associated animal size.

**Figure 3:**
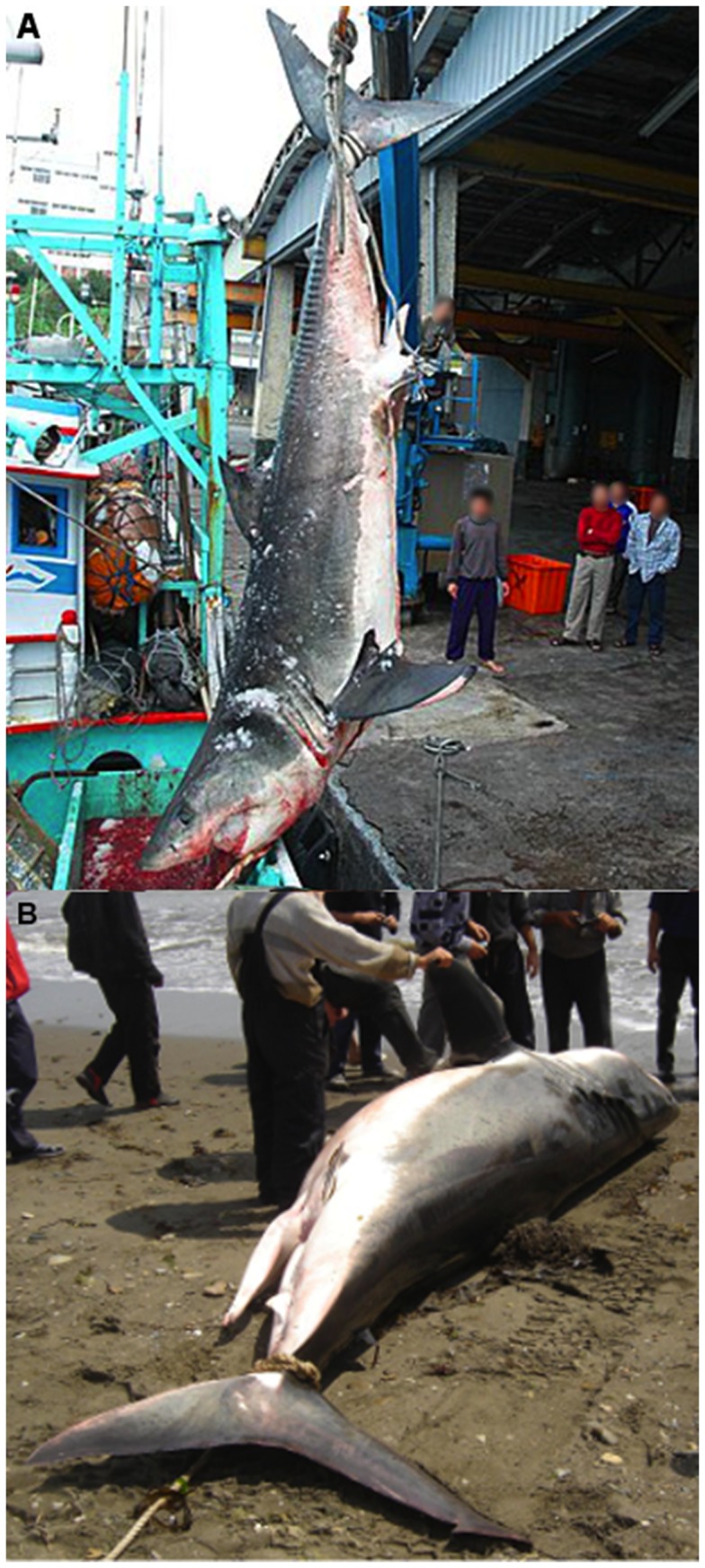
Male white shark. A) Male captured on February 17, 2009 in Taitung, Taiwan measuring 500 cm total length and weighing 1020 kg. Photo obtained from: http://tw.myblog.yahoo.com/jw!duL4dwaTBB7FYwm6Q0vtIm8d/gallery?cfid=144&act=&fid=144&nfid=&yuid=jw!duL4dwaTBB7FYwm6Q0vtIm8d&page=1&.crumb=8mdZj9IAdn6 B) Male captured on July 19, 2007 in Aniva Bay Sakhalin, Russia measuring 504 cm total length and weighing 1111 kg.

**Figure 4:**
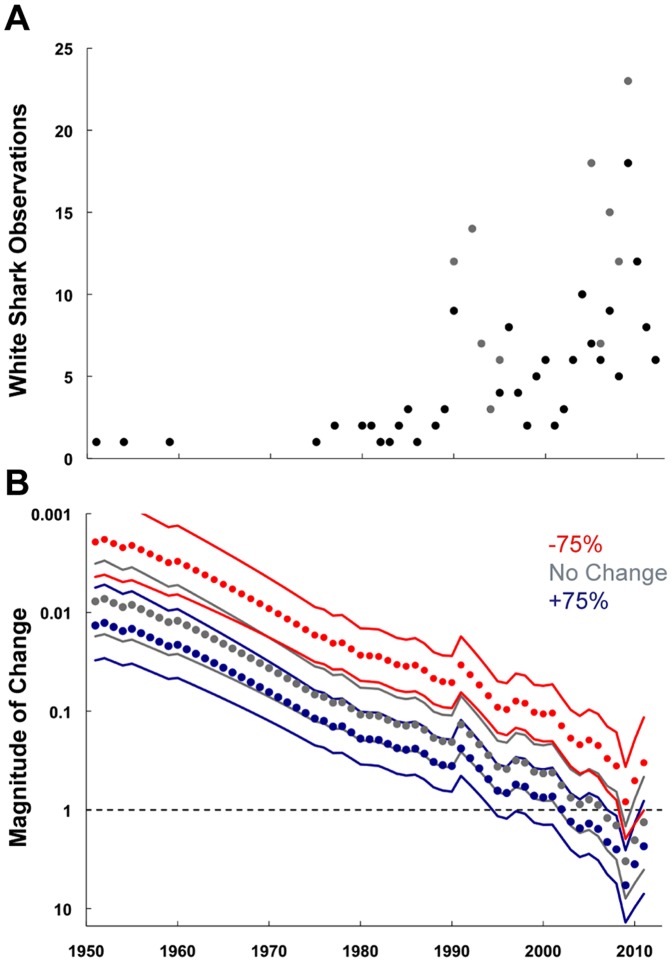
Population trend analysis. A) White shark observations by year. Black circles indicate data included in population trend analysis. Gray circles indicate data from focused monitoring in Japan that was excluded prior to undertaking the analysis. B) Estimates of changes in relative abundance for any reference year between 1951–2011 under different assumptions of trends in observation effort.

**Figure 5:**
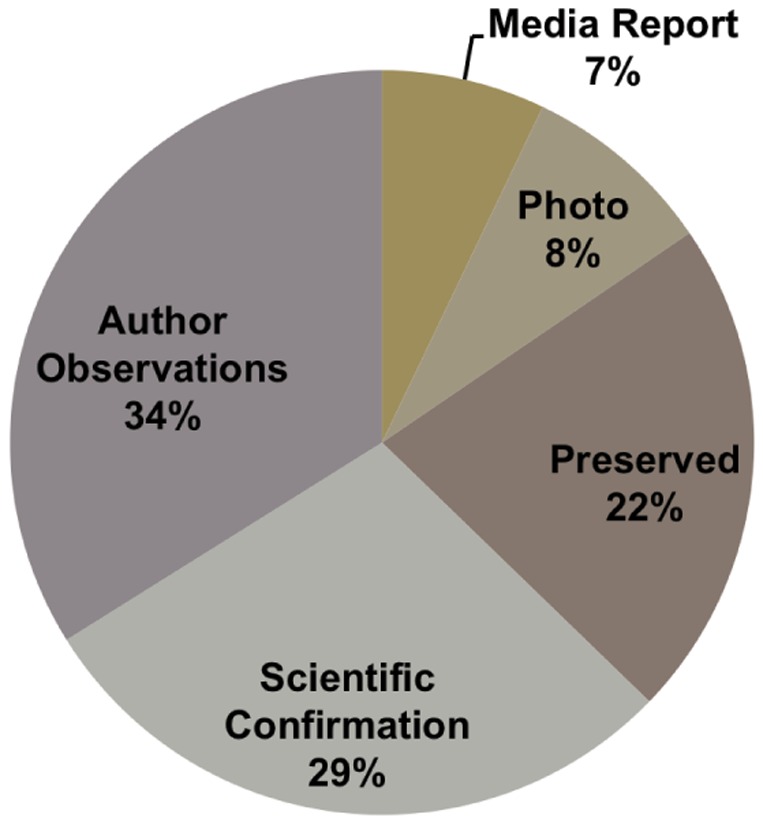
Method of confirming white shark observations. Data are shown as a percentage; Media Report - from newspapers or online news sources; Photo - confirmed by pictures of the individual shark (either through personal communications or online but not through news source); Preserved - individuals or remains of an individual, i.e. jaws that are held in a personal or museum collection; Scientific Confirmation - observations that have been previously reported in the scientific literature; Author Observations - personal observations or communications by the authors.

A total of 248 white shark observations were documented; after the removal of duplicates 240 reliable observations remained (Table S1). Records of white sharks were found in seven countries ranging from the most northerly in temperate waters off Russia to the most southerly in tropical waters off Vietnam (Fig. 2A, 3), spanning a straight line latitudinal distance of 4,300 km. Location of landing was recorded for 169 observations, while the remaining reports only included the country of landing (Fig. 2A, 2B). Observations of white sharks occurred between 1951 and 2012; the year of capture was recorded for 212 animals. The number of observations increased over time, with two peaks occurring in 1992 and 2009 and no observations documented between 1960 and 1974 (Fig. 4A). Correct species identification for the 240 white sharks was confirmed through records from trained scientists, personal observations, preserved remains, photographs or media reports (Fig. 5; Table S1). The method of capture was documented for 79 observations with 56 animals caught in set nets, 12 by set lines, 3 each by either gillnet, seine or trawls and one each by harpoon and crab basket (Table S1). The majority of these captures occurred in Japan (76) with 25 occurring in the set net fishery off Ibaraki and 11 in the set line fishery surrounding Okinawa. Of these captures, most sharks were caught in spring (April–June) (38), followed by winter (January–March) (18), summer (July–September) (12), and autumn (October–December) (10).

**Figure 6:**
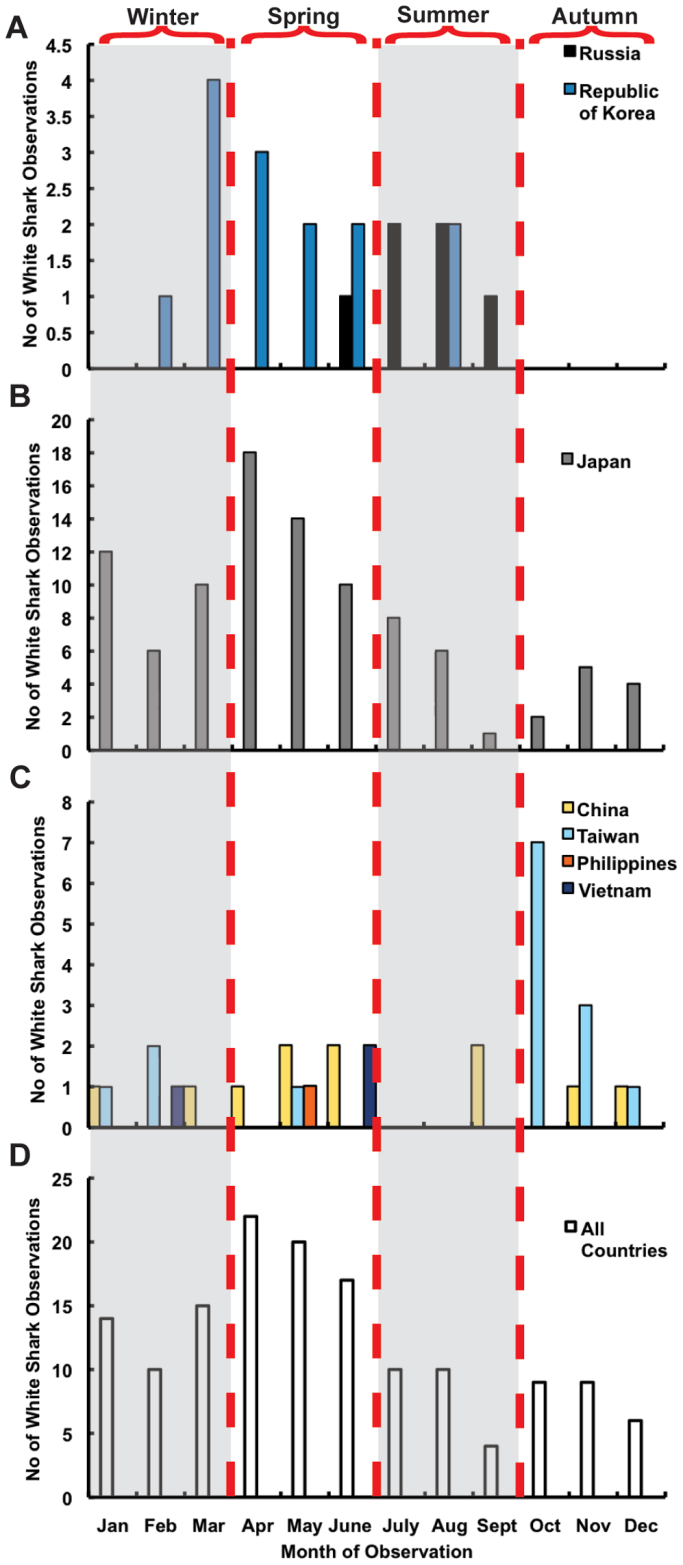
White shark observation by month and latitude. A) Republic of Korea (blue bars) and Russia (black bars) B) Japan (gray bars) C) China (yellow bars), Taiwan (light blue bars), Vietnam (dark blue bars), and the Philippines (orange bars), D) All observations combined. Winter and summer months are shaded gray.

When considering all regional observations, month of capture was reported for 146 animals (Fig. 6). White sharks were reported across all months and seasonal trends mirrored those of documented fishery captures in Japan with spring having the highest number of observations (59), followed by winter (39), summer (24), and autumn (24). In the more northern countries (Russia and the Republic of Korea) white sharks were not observed in autumn and early winter from October–January (Fig. 6A). For Japan, white sharks were observed across all months of the year with a peak between April–May (Fig. 6B). In the most southerly countries (China, Taiwan, the Philippines, and Vietnam) white sharks were observed in all months except during summer (July–August) (Fig. 6C).

There were two time periods where focused white shark monitoring occurred in Japan (1990–1994 and 2005–2009). To avoid any biases caused by increased observation effort these data points were removed prior to population trend analysis. When accounting for no change in observer effort over time, the population trend estimate indicated that the relative abundance of white sharks has been stable or increasing in the region until recently (2007) (Fig. 4B). Parameterization of a 75% decrease in observer effort indicated the relative abundance of white sharks has remained stable or increased throughout the reference period, while a 75% increase in observer effort indicated a minor decline in relative abundance since 2002.

**Figure 7:**
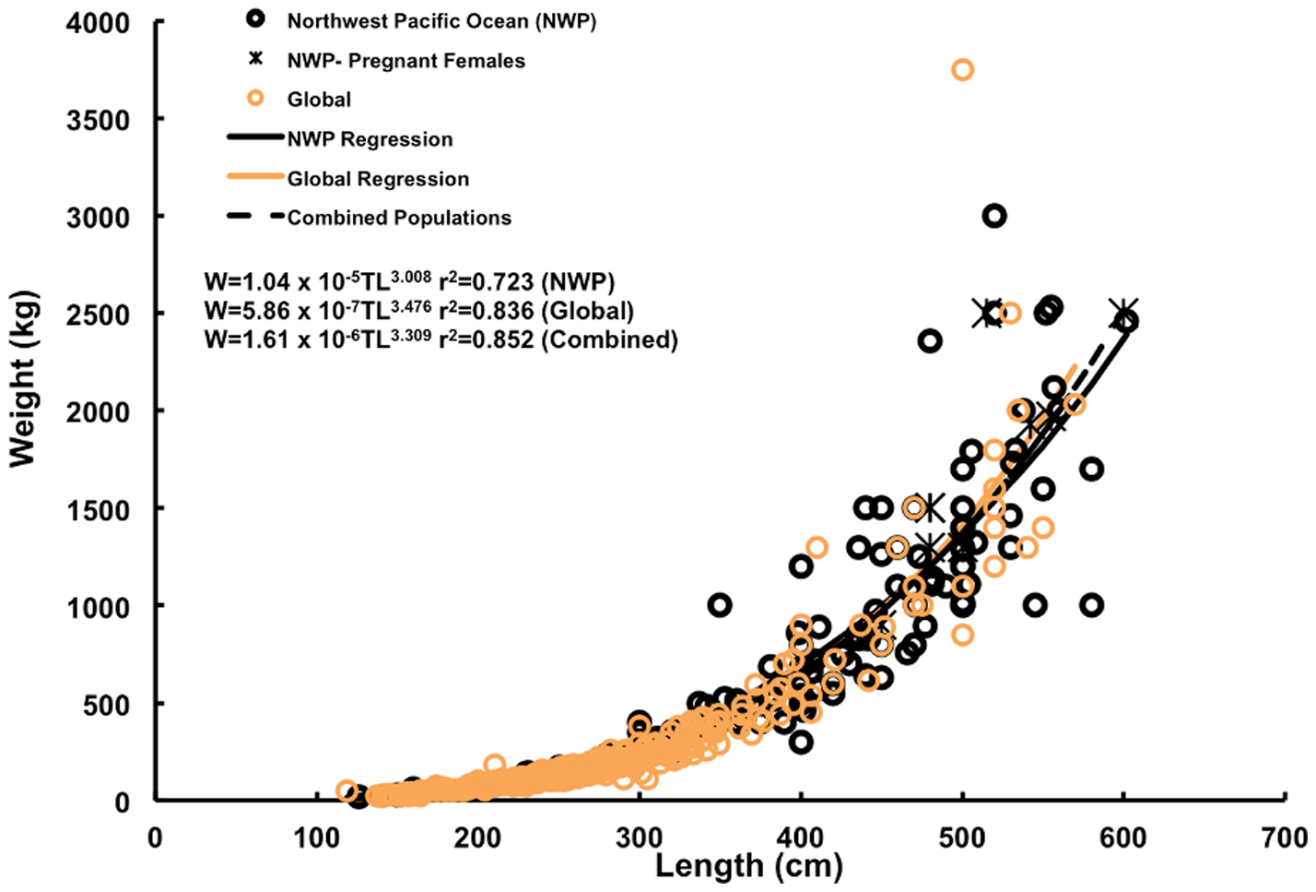
Length-weight regressions for white sharks by region. Regressions were fit to white shark data for the Northwest Pacific (black circles/line), global data recorded by trained scientists (orange circles/line) and all data combined (dashed black line). Pregnant females from the Northwest Pacific (black stars) were not included in the analysis.

Size of animal (TL) was recorded for 175 observations (Fig. 2C; Table S1). For this study, sharks whose weight and total length were estimated are identified in Table S1. The smallest recorded shark measured 126 cm TL and was caught in Primorye, Russia, while the largest shark was landed in Seven Star Lake, Taiwan and was estimated to be 670–700 cm TL (Fig. 2C). The largest accurately measured shark was caught in the East China Sea and measured 602 cm TL. White sharks <200 cm TL were observed in Russia (n = 1)(September), the Republic of Korea (1), Japan (2) (July) and Vietnam (1) (June). Weight was measured or estimated for 162 white shark observations (Table S1). The lowest weight recorded was 16 kg (for the 126 cm TL specimen-Primorye, Russia), while the largest estimated weight was 3000 kg for a 520 cm TL shark caught in Hikari City, Japan. The most reliable weight for the heaviest animal documented was 2530 kg, a 555 cm TL shark that was recorded by a fishery wholesaler in NingPuo, China. There was no significant difference in the slope of the length-weight regression between females and males, so data were combined (interaction of length and sex: *F* = 0.3 and *P* = 0.59). The intercept for females was higher than males indicating that females grow larger than males. When testing between combined sex length-weight data for the Northwest Pacific and global data, no significant difference between slopes was detected (interaction of length and region: *F* = 0.06 and *P* = 0.81). The relationship between length and weight was expressed as: Northwest Pacific W = 1.04e^−5^TL^3.008^ (*r^2^* = 0.72), global data W = 5.86e^−7^TL^3.476^ (*r^2^* = 0.84). A regression for all data was calculated as W = 1.61e^−6^TL^3.309^ (*r^2^* = 0.85) (Fig. 7).

**Figure 8:**
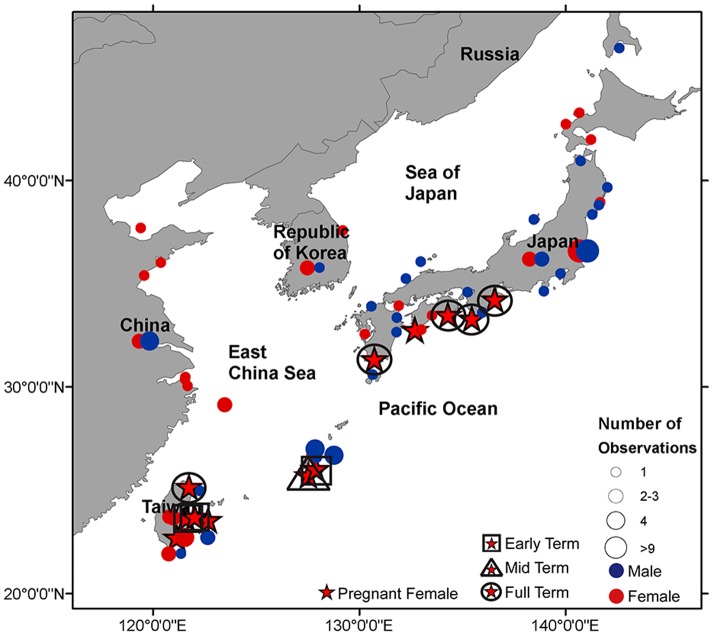
Location of white shark observations by sex. The occurrence of male, female and pregnant female white sharks in the Northwest Pacific. Circles on land underneath country label indicate observations that did not report a specific landing location. For pregnant females, black squares indicate early term, black triangles indicate mid term and black circles indicate full term for individuals where embryo size was reported.

**Figure 9:**
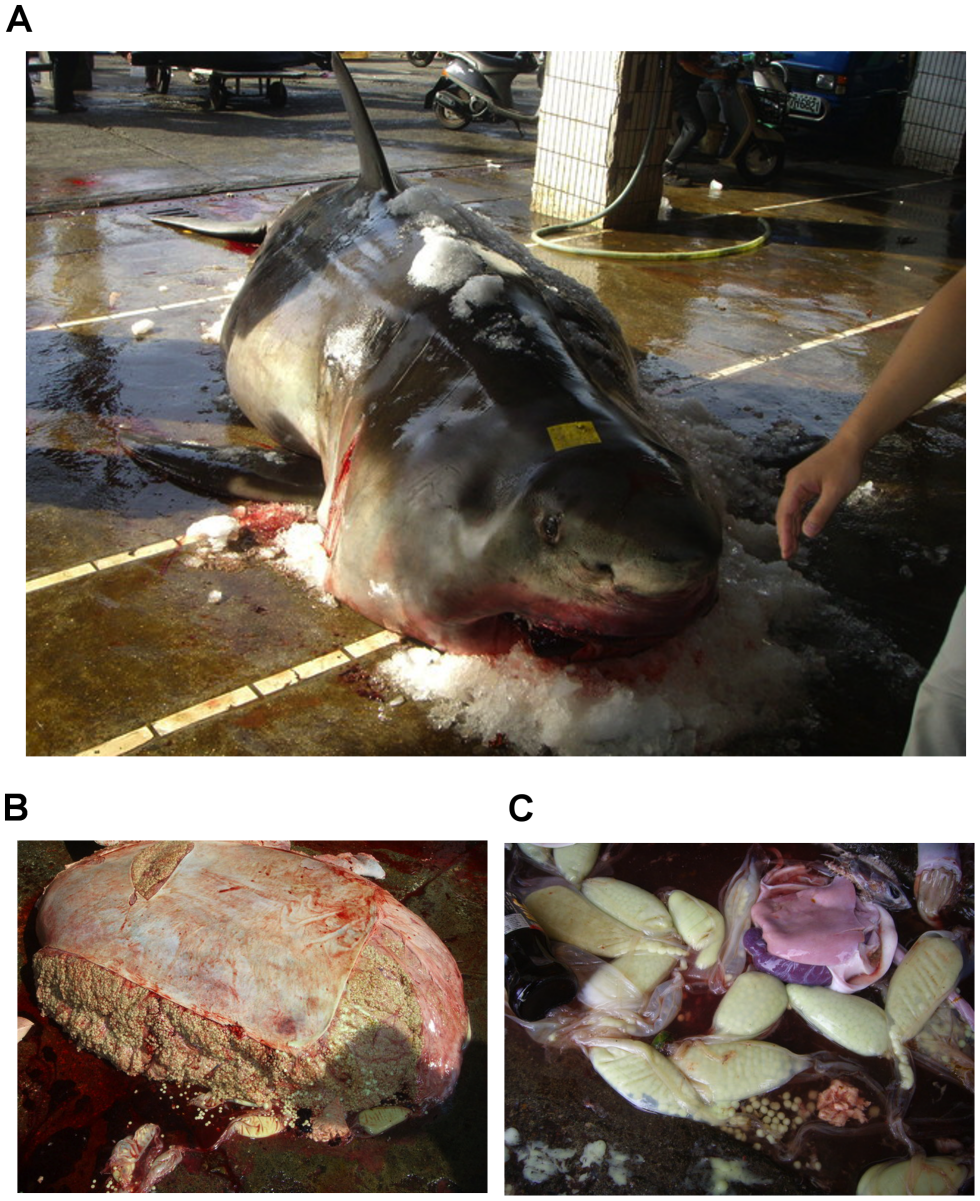
Pregnant white shark. A) Pregnant female captured on November 7, 2008 in Southern Bay Dock, East Taiwan, measuring 542 cm total length and weighing 1930 kg B) Ovary C) Egg cases measuring 10 cm. Photos obtained from: http://tw.m.wretch.yahoo.com/album/redo0905/12.

Sex was recorded on 113 occasions. A total of 53 males and 60 females were documented (Fig. 8; Table S1) of which 11 (18.3%) were pregnant and one individual was suspected to be pregnant (Fig. 9; Table 2). The observed sex ratio was not significantly different from 1∶1 for all countries combined (1∶1.1, *Χ^2^* = 0.43, p = 0.51) or individually China (1∶2.8, *Χ^2^* = 3.3, p = 0.07), Japan (1∶0.7, *Χ^2^* = 2.3, p = 0.13) and Republic of Korea (1∶3, *Χ^2^* = 1.0, p = 0.32), while it was significantly biased towards females for Taiwan (1∶4, *Χ^2^* = 7.2, p<0.01). Sex was reported for one shark in Russia (male) and for none in Vietnam and the Philippines.

**Table 2:** Description of pregnant white sharks in the Northwest Pacific Ocean and Worldwide.

Date	Location	TL (cm)	Weight (kg)	Number of Embryos	Embryo Size (cm TL)	Source
Feb. 16, 1985	Kin, Japan	555	1970	0	egg cases	Uchida et al. [32], Uchida et al. [35]
Apr. 2, 1986	Taiji, Japan	470	—	7	100–110	Uchida et al. [32], Uchida et al. [35]
May 14, 1992	Uchinoura, Japan	480	1500	5	130	Nakaya [33], Uchida et al. [35]
May 22, 1992	Toyo, Japan	515	2500	10	135–151	Nakaya [33], Uchida et al. [35]
Mar. 30, 1994	Otuki-Machi, Japan	480	1500	3	140	Author Observation
May 18, 1997	Shiura, Minamijima-machi, Japan	500	1300	5	—	Author Observation
Jan. 18, 2007	Okinawa, Japan	480	1300	7	mid-term	Author Observation
Feb-March 1988	Keelung,Taiwan	—	—	3	100	D. Ebert p.c.
May 1997	Seven Star Lake, Taiwan	670–700	2500		—	Author Observation
Oct. 13, 1997	Baisolian, Taiwan	—	2000	8	—	Author Observation
Nov. 7, 2008	East Taiwan	542	1930		10 and egg cases	Author Observation
Nov. 2, 2012	Taiwan	450	900	6	20	Author Observation
Nov. 13, 1991	North Cape, New Zealand	536	—	7	143–145	Francis [17]
Nov. 17,1981	Queensland, Australia	320*	—	4	—	Paterson [86]
Nov. 26, 1982	Queensland, Australia	400*	—	11	—	Paterson [86]
Nov. 26,1982	Queensland, Australia	420*	—	14	—	Paterson [86]
Mar. 1, 1994	S. Australia	—	—	2	127	JD Stevens p.c.
Nov. 9, 2003	Aaiheke Island New Zealand	—	—	3–5	150	New Zealand Herald
Oct. or Nov.	South Australia	520	—	6–7	30	Bruce [87]
Oct. or Nov.	South Australia	470	—	13	5	Bruce [87]
Oct. or Nov.	South Australia	420	—	11	60	Bruce [87]
Aug. 1, 1976	Skonnet RI,USA	—	—	—	122	Richard Ellis p.c.
July 16, 1996	Malindi, Kenya	—	—	7–17	110	Cliff et al. [65]
Summer 1934	Alexandria, Egypt	430	—	9	61	Norman & Fraser [88], Ellis & McCosker [89]
Sept. 1, 1992	Cape Bon, Tunisia	>500	—	2	Full-term	Fergusson [27]
Feb. 26, 2004	Gulf of Gabes, Southern Tunisia	—	—	4	132–135	Saïdi et al. [71]

p.c.- personal communication.

*Francis 1996 suggests incorrect measurements or not TL.

**Figure 10:**
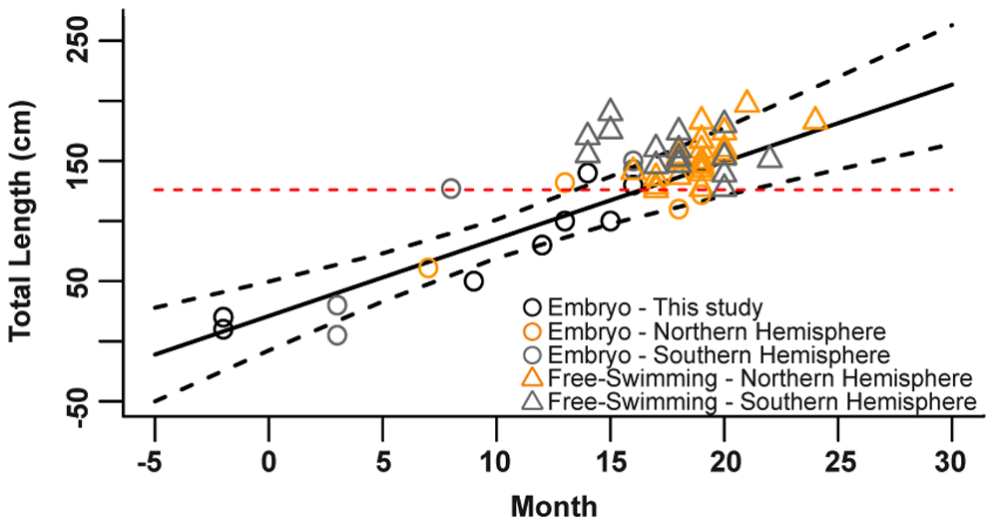
Month of observation and size of white shark embryos and free-swimming juveniles worldwide. Data are for white shark observations in this study and globally. Month zero is January in Northern Hemisphere and July in Southern Hemisphere. For unborn animals, black circles indicate embryos from this study (Northwest Pacific; n = 9); orange circles indicate embryos from the Northern Hemisphere (n = 4), while gray circles indicate embryos from the Southern Hemisphere (n = 5). For free-swimming juvenile sharks, orange triangles indicate individuals from the Northern Hemisphere (n = 18), and gray triangles indicate individuals from the Southern Hemisphere (n = 17). The solid black line indicates the linear regression for embryos from this study, while the dashed black lines indicate the confidence intervals. The red dashed line indicates size at birth.

Pregnant females ranged in size from 450–600 cm TL and contained an average of six embryos (ranging from egg cases to 10 embryos) (Table 2). Egg cases were found in females captured in early autumn, mid-term embryos were present in winter, and full-term embryos (130–150 cm TL) were present in spring (Table 2). The relationship between embryo size and month of capture predicted a gestation period of 20 months (n = 9, slope = 6.4 cm/month, SE = 1.0 cm/month, p<0.01, r^2^ = 0.84) (Fig. 10). Pregnant white sharks were geographically separated during the various stages of gestation; egg cases and early term embryos were present in individuals from the southern locations of Taiwan and Okinawa, whereas full term embryos were found in individuals mostly captured near mainland Japan.

## Discussion

Determining the spatial and temporal distribution of threatened species is a critical first step for effective conservation measures. This is the first study to collate and present observations of white sharks in the Northwest Pacific Ocean in order to define the geographic extent of the population. While we do not assume that we obtained all records in the region over the past 60 years, these observations provide important baseline data for this little known population, providing a benchmark to instigate further research for population-specific conservation and management efforts.

The observations of white sharks in the Northwest Pacific increased over time and showed a bimodal trend that coincided with focused monitoring of catches (1990–1994; [33]) and sampling of white sharks by Ibaraki Prefectural Oarai Aquarium staff (2005–2009; [36]) in Japan, and consequently represent a bias in observed trends. Aside from these two periods, there was no formal monitoring program of white shark catches and actual observations may be higher due to fishermen releasing individuals at sea and/or not reporting catches [33]. The number of observers reporting large white sharks in this region have likely increased over time due to a combination of improved fishing gear/effort and increasing media coverage due to technological advances and heightened public interest in large marine predators. However, smaller individuals likely do not elicit the same media response and catches may go unreported or may be misidentified with other species in the family Lamnidae, resulting in lower observation numbers for this size class [45]. Additionally, during the study period there was no requirement to report catches of white sharks, which may have affected observer effort. When accounting for an increase, no change, and decrease in observer effort over the study period within the generalized linear model, minimal effects of observer effort on model results were observed for all scenarios. Overall the population trend analysis, which identifies changes in relative abundance and not absolute population estimates, found that the Northwest Pacific population was relatively stable. Evidence for a recent declining trend in relative abundance of sharks with 75% increase in observer effort, identifies that it is important to enact ongoing monitoring to detect if this decline continues. Previously this analysis has documented an increase in white shark abundance off the coast of Massachusetts [30], and steep declines in relative abundance of white shark in the eastern Adriatic and eastern Canada [42].

There are no directed fisheries for the relatively rare white shark; however, bycatch in artisanal and commercial fisheries has been reported from the coast of California [46], Mexico [47], Australia [48], and in beach protection programs off Australia [48] and South Africa [49]. In the current study, the majority of the animals categorized as bycatch were between 300–580 TL, which contrasts with data from the above regions where most animals were young-of-the-year or juveniles (<300 cm TL) [46]–[48]. It has been proposed that larger white sharks (>300 cm TL) are less susceptible to entanglement in fishing gear [46], [49], but this finding is not supported by our data for the Northwest Pacific. Given that in this study the largest numbers of white sharks documented as fisheries bycatch were caught in Japan, specifically the set net fishery off Ibaraki, further research including biological sampling, could shed light on this discrepancy and other related questions of regional population size and distribution.

Temperatures in this region vary both seasonally and geographically, ranging from 0°C at northern latitudes off Russia to 28°C off Vietnam [50], [51]. White sharks were not observed during autumn and early winter in the most northerly latitudes, similar to the absence of white sharks in the northern latitudes of the Northeast Pacific [52]. Similarly, sharks were absent from the most southern latitudes during July-August, suggesting they have a preferred temperature niche. White sharks have been documented in water temperatures from 3–27°C [13]–[15], [40], [52], [53]; but typically spend most of their time in a narrow range of water temperatures, from 16.4–24.7°C [47], [54]–[57]. As the SST off Japan encompasses these temperature ranges [58], [59], it is possible that sharks move along the Japanese coastline concurrent with the seasonal change in SST. Alternatively, white sharks may inhabit these temperatures due to prey availability [57]. While there are documented seal rookeries along the Japanese coastline [60], [61] further research is required to determine the extent white sharks utilize them as a food source. Regardless of the cause of habitat preference (temperature or prey availability) these data suggest that Japan is an important aggregation site for the regional population similar to Central California and Guadalupe Island in the Northeast Pacific [15], [62].

Prior to this study, the largest white shark on record worldwide was reported to be 600 cm TL and was caught in Western Australia [12]. One shark in this study was estimated to be 670–700 cm TL; however, examination of the jaw dimensions revealed it to be smaller (approximately 600 cm TL; Author Observation). The longest reliably measured shark in this study now represents the largest white shark on record worldwide (602 cm TL); this shark was measured by a fish factory owner following instructions from one of the authors (see Fig. S1). Fishermen commonly exaggerate when estimating the size of large fish [12], [33], which may result in a bias in this and other observation data; however, the length-weight data from the Northwest Pacific Ocean (including estimated lengths and weights) agreed with global data recorded by trained scientists providing confidence in the quality of the data. This study reports some of the heaviest white sharks on record and consequently adds important data to the upper end of the length-weight relationship for this species (KwaZulu-Natal Sharks Board, unpublished data) [17], [27], [43].

Single regional nursery grounds for white sharks have long been hypothesized [15], [55], but recent genetic and tracking data indicates the presence of multiple nursery grounds off Eastern Australia [63]. Similarly, individuals <200 cm TL in this study were geographically widespread. Assuming that these small juveniles have relatively restricted home ranges [64], our data suggests the occurrence of multiple nursery grounds in the Northwestern Pacific with associated implications for management. White sharks of all sizes were observed throughout the region, suggesting either a lack of size segregation or regionally structured populations. In South Africa, sub-adults and small adults aggregate at pinniped colonies and make offshore migrations [13], [55], while larger adults, including pregnant females, are typically found in the tropical waters of the Western Indian Ocean [65]. Similarly in the Northeast Pacific, size segregation occurs and adult sharks aggregate to one of two locations (Central California or Guadalupe Island) and make defined offshore migrations [15]. White sharks have also been documented to use specific habitats by size within aggregation sites [66]. Accepting that observation data provides only a snap shot of a species’ distribution and movement, it is likely, that size segregation occurs in the region but the coarseness of the observation data is unable to determine this.

Conclusions about sexual segregation by season or location could not be made due to under-reporting of sex. Similar to size segregation, it is possible that fine-scale sexual segregation may be occurring within the Northwest Pacific white shark population, but tracking data is required to confirm this. Sexual segregation in white sharks has been described in the Northeast Pacific Ocean, whereby males make annual migrations and large likely pregnant females migrate biannually, remaining in offshore waters for a prolonged period of time [15], [40]. In the Neptune Islands, South Australia, females are only observed from autumn to mid-winter while males are present year round [64]. Conversely in False Bay, South Africa, females are present year-round while males are only present during autumn and winter [67]. Because fishing pressure can affect one sex unequally in sexually segregated populations [68], it is important to determine the extent of sexual segregation occurring in the region.

Of the 11 pregnant females documented from the Northwest Pacific, four were described previously [32], [33], [35] and there was one additional suspected pregnant female (Table 1). The geographic segregation of these pregnant individuals by gestation stage may suggest that pregnant females have variable habitat preferences during this period. Previous opportunistic sampling of white sharks has allowed the description of the reproductive system in mature males [69] and pregnant females [32], [33], [35]; however, little is known about the migration patterns of pregnant females. For the Northeast Pacific, it is thought that females remain offshore during gestation and return to inshore nurseries to pup [15], [70]. This hypothesis is supported by data from other regions, with near-term females being captured nearshore in Kenya [65] and Tunisia [71]. The estimated 20-month gestation period for sharks in the Northwest Pacific is slightly longer than the previously suggested 18-month gestation period [15], [44], [70]. The spring (April–June) parturition in the Northwest Pacific, is slightly before the late spring – mid-summer (May–August) parturition found in the Northeastern Pacific [15], and earlier (assuming offset by 6 months) from the southern hemisphere South Australia parturition, which is purported to occur in summer – mid-autumn (December–May) [48].

## Conclusions and Recommendations

White sharks are protected globally by a variety of measures (Table 1); however, participation in these agreements varies in the Northwest Pacific. Regional management units may provide a mechanism to preserve genetic diversity and protect distinct population segments [4]. This highlights the need for improved regional protection of white sharks. International cooperation among countries throughout the Northwest Pacific is required to establish a management agenda, initiate systematic monitoring and biological sampling programs, and to reach agreement over commitments to international conventions [72]. Regional workshops that involve various stakeholders (i.e. policy makers, scientists, and local fisherman) should be conducted to determine the best practices to accommodate local customs and requirements [73]. Formal monitoring programs such as the recently enacted regulation in Taiwan requiring fishermen to report catches of white sharks of all size classes [74], if properly implemented and enforced, would allow for a more accurate assessment of population trends in the region and help to determine aggregation sites.

There are several broad areas of scientific research that should be conducted to promote conservation [1]. Satellite tagging studies can provide details on migration (by size and sex) [40], [41], aggregation sites, and potential nursery grounds [16], [22], while acoustic tagging can give fine-scale details on localized movements during residency periods [62], [67]. Identifying aggregation sites will provide locations to conduct biological sampling to better understand the role of white sharks in regional food webs and continue population assessments. Chemical tracer techniques can be successfully used to infer the diet and trophic ecology of these large predators [75], [76] and hormone analysis, for example, can elucidate information on reproductive status [77]. Combining photographic identification with mark recapture or acoustic tracking data can be used to quantitatively investigate population trends [21], [78]. In addition, identification of aggregation sites can facilitate economically profitable shark-based ecotourism [79], [80]. While not without concern over possible harmful effects on ecosystems, white shark cage diving operations at aggregation sites in Australia, Mexico, New Zealand, South Africa, and the US yield financial benefits for operators and associated communities as well as opportunities for education and research [79].

Mitochondrial DNA analyses of Japanese white sharks indicate these individuals form a monophyletic clade separate from other geographic regions including the Northeast Pacific [36]. Furthermore, of more than 200 white sharks satellite tagged in the Northeast Pacific [14], [40], [70], [81]–[85], no animals have yet undertaken trans-oceanic migrations to the Northwest Pacific region. This, coupled with the large number of observations in this study across the entire Northwest Pacific and the relatively stable abundance of animals over the past 60 years, support the occurrence of a distinct sub-population of white sharks, which is widely dispersed from northern temperate to southern tropical latitudes.

## Supporting Information

Figure S1 **White shark total length measuring protocol.**
(TIF)

Table S1 **Records of White Shark Observations in the Northwest Pacific Ocean 1951–2012.**
(DOC)

Table S2 **Global records of white shark embryos and free-swimming juveniles.**
(DOC)
