# Supplementary figures and images for: The Last Frontier: Catch Records of White Sharks (Carcharodon carcharias) in the Northwest Pacific Ocean
Source: PLoS One. 2014 Apr 16;9(4):e94407. doi: 10.1371/journal.pone.0094407 (PMC3989224; doi:10.1371/journal.pone.0094407)

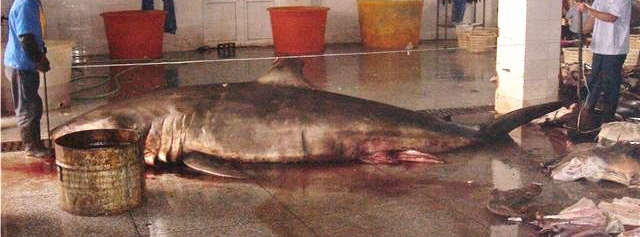

Supplement: Figure S1 — White shark total length measuring protocol. (TIF) [file pone.0094407.s001.tif]
