# Supplementary material for: The Last Frontier: Catch Records of White Sharks (Carcharodon carcharias) in the Northwest Pacific Ocean
Source: PLoS One. 2014 Apr 16;9(4):e94407. doi: 10.1371/journal.pone.0094407 (PMC3989224; doi:10.1371/journal.pone.0094407)
Supplement: Table S1 — Records of White Shark Observations in the Northwest Pacific Ocean 1951–2012. (DOC) [file pone.0094407.s002.doc]

**Table S1- Records of White Shark Observations in the Northwest Pacific Ocean 1951-2012**

| **Date** | **Country** | **Location of Landing** | **Latitude** | **Longitude** | **Length (cm TL)** | **Weight (kg)** | **Sex** | **Fishing Method** | **Source** | **Source** |
| --- | --- | --- | --- | --- | --- | --- | --- | --- | --- | --- |
|  | China | TsingTao | 36.02 | 120.39 | 400* |  |  |  | preserved | Jaws preserved in a museum in TsingTao |
| 1995 | China |  |  |  | 450.00 | 1500 | M |  | personal communication | Huang personal communication |
| 1996 | China | GuangZhou | 21.87 | 113.46 |  | 2950* |  |  | preserved | Jaws preserved. Huang personal communication |
| 1999 | China |  |  |  |  | 500 |  |  | media report | Media report |
| 1999 | China |  |  |  |  | 2200 |  |  | personal communication | Huang personal communication |
| November 1, 2001 | China | East Sea | 29.15 | 123.49 | 580.00 | 1000* | F |  | preserved | Mounted in ZheJiang Natural History Museum |
| September 10, 2002 | China | Northern Yellow Sea | 38.84 | 123.02 | 337.00 | 500 |  |  | preserved | Mounted at a local museum in Daliang |
| 2002 | China | East Sea | 29.15 | 123.49 | 602.00 | 2460 | F |  | personal communication | Author observation |
| 2003 | China | TsingTao | 36.02 | 120.39 | 480.00 | 2360 |  |  | personal communication | Author observation |
| 2004 | China |  |  |  |  | 500* | F |  | personal communication | Author observation |
| 2004 | China |  |  |  |  | 1400 |  |  | personal communication | Wen personal communication |
| 2004 | China |  |  |  | 500* | 1700 |  |  | photo | Photos of being dissembled shown on internet |
| 2005 | China | Dalian | 38.82 | 121.66 |  | 800 |  |  | preserved | Mounted for museum display |
| 2005 | China | NingPuo (Ningbo) | 30.06 | 121.69 | 555.00 | 2530 | F |  | preserved | Author observation |
| April 18, 2006 | China | JaingSu County | 33.09 | 121.29 | 466.00 | 755 |  |  | personal communication | Author observation |
| 2006 | China |  |  |  |  | 600 |  |  | personal communication | Huang personal communication |
| 2007 | China | Rizhao, Shandong | 35.40 | 119.59 | 450.00 | 630 | F |  | media report | http://english.people.com.cn/200705/09/eng20070509_373174.html |
| 2007 | China |  |  |  |  | 1950* | F |  | personal communication | Author observation |
| 2007 | China |  |  |  |  | 800* |  |  | personal communication | Li personal communication |
| 2007 | China |  |  |  | 468.00 | 1080 | M |  | personal communication | Li personal communication |
| May 7, 2007 | China | ZiZhau, ShanDong | 37.70 | 119.42 | 450.00 | 1260 | F |  | media report | Media report |
| June 12, 2007 | China | TsingTao | 36.02 | 120.39 | 520* | 1200* | F |  | photo | Author observation |
| March 15, 2008 | China | ZhongChen, ShanDong | 30.37 | 121.57 |  | 2500 | F |  | personal communication | Author observation |
| June 10, 2008 | China | Zhejiang | 30.47 | 121.59 | 550.00 | 1600 | F |  | personal communication | Author observation |
| 2009 | China |  |  |  | 530.00 | 1460 | M |  | preserved | Jaws preserved |
| 2009 | China |  |  |  | 473.00 | 1250 | M |  | personal communication | Huang personal communication |
| 2009 | China |  |  |  |  | 800 |  |  | personal communication | Li personal communication |
| 2009 | China |  |  |  | 300* |  |  |  | photo | Photo of mount displayed on street |
| January 19, 2009 | China |  |  |  |  | 600 |  |  | photo | Photos of being dissembled on street |
| September 15, 2009 | China |  |  |  | 460.00 | 1300 |  |  | personal communication | Li & Tsai personal communication |
| May 4, 2010 | China | RauShan, ShanDong | 36.62 | 121.63 |  | 1500* |  |  | photo | Photos of being displayed on street |
| December 10, 2010 | China |  |  |  |  | 1600 | F |  | personal communication | Li personal communication |
|  | Japan |  |  |  |  |  |  |  | preserved | Mounted at National Museum of Nature and Science |
|  | Japan |  |  |  |  |  |  |  | preserved | Jaws preserved in unknown museum |
|  | Japan |  |  |  |  |  |  |  | preserved | Jaws preserved |
|  | Japan |  |  |  |  |  |  |  | preserved | Jaws preserved in unknown museum |
|  | Japan |  |  |  |  |  |  |  | preserved | Head preserved in Kesennuma Fish Market |
|  | Japan |  |  |  |  |  |  |  | preserved | Mounted at Kanagawa County Aquarium |
|  | Japan |  |  |  |  |  |  |  | preserved | Preserved in Minamichita Aquarium |
|  | Japan |  |  |  |  |  |  |  | preserved | Preserved in Churarumi Aquarium, Okinawa |
|  | Japan |  |  |  |  |  |  |  | preserved | Jaws preserved |
|  | Japan |  |  |  |  |  |  |  | preserved | Preserved head in Himeji-jo Municipal Aquarium |
|  | Japan |  |  |  |  |  |  |  | preserved | Head mounted for display at Oarai mobile aquarium |
|  | Japan |  |  |  |  |  |  |  | preserved | Mounted at Yamahaku County Museum |
|  | Japan |  |  |  | 150.00 |  |  |  | preserved | Jaws preserved |
|  | Japan |  |  |  | 400.00 | 540 | M |  | preserved | Mounted at Ibaraki Oarai Aquarium |
|  | Japan |  |  |  | 400* |  |  |  | preserved | Skin mounted in Ibaraki Oarai Aquarium |
|  | Japan |  |  |  | 400.0* |  |  |  | preserved | Jaws preserved Churaumi Mobile Aquarium, Okinawa |
|  | Japan |  |  |  | 420.00 |  |  |  | preserved | Mounted at Sakaiminato cultural museum |
|  | Japan |  |  |  | 450* |  |  |  | preserved | Jaws preserved in Ibaraki Oarai Aquarium |
|  | Japan |  |  |  | 450* |  |  |  | preserved | Jaws preserved in Ibaraki county Natural Museum |
|  | Japan |  |  |  | 450* |  |  |  | preserved | Jaws preserved in Hakan Aquarium Okinawa |
|  | Japan | Kyushu | 32.57 | 130.27 | 480.00 |  | F |  | personal communication | Author observation |
|  | Japan |  |  |  | 500.00 |  |  |  | preserved | Head and fin preserved in Hokkaido University |
|  | Japan |  |  |  | 500.00 | 1300 | F |  | preserved | Mold of specimen kept in Rias Shark Museum, Kesennuma |
|  | Japan |  |  |  | 500.0* | 1200* | M |  | photo | Author observation |
|  | Japan | Aomori | 40.95 | 140.76 | 500.0* | 1000* |  |  | preserved | Author observation |
|  | Japan |  |  |  | 550* |  |  |  | photo | Author observation |
| October 2, 1954 | Japan | Oomura Bay, Nagasaki | 32.90 | 129.88 |  | 2000 |  |  | media report | K. Nakaya; San Diego Union, 105/1954 |
| November 21, 1975 | Japan | Motobu, Okinawa | 26.66 | 127.87 | 390.00 | 400 | M | set line | scientific confirmation | Uchida et al. 1996 [1] |
| January 18, 1977 | Japan | Motobu, Okinawa | 26.66 | 127.87 | 360.00 | 400 | M | set line | scientific confirmation | Uchida et al. 1996 [1] |
| January 18, 1977 | Japan | Motobu, Okinawa | 26.66 | 127.87 | 400.00 | 500 | M | set line | scientific confirmation | Uchida et al. 1996 [1] |
| March 5, 1980 | Japan | Motobu, Okinawa | 26.66 | 127.87 | 471.00 | 1000 | M | set line | scientific confirmation | Uchida et al. 1996 [1] |
| June 26, 1981 | Japan | IE Island, Okinawa | 26.70 | 127.79 | 506.00 | 1790 | F | set line | scientific confirmation | Uchida et al. 1996 [1] |
| July 9, 1982 | Japan | Sainonada, Houjou City, | 34.03 | 132.81 | 460.00 |  |  | set net | media report | Asahi News Paper |
| November 15, 1984 | Japan | IE Island, Okinawa | 26.70 | 127.79 | 400.00 | 680 | M | set line | scientific confirmation | Uchida et al. 1996 [1] |
| February 16, 1985 | Japan | Kin, Okinawa | 26.44 | 127.91 | 555.00 | 1970 | F† |  | scientific confirmation | Uchida et al. 1996 [1] |
| May 30, 1985 | Japan | Todohokke | 41.97 | 141.24 | 530.00 | 1300 | F | set net | scientific confirmation | Nakano and Nakaya 1987 [2] |
| May 31, 1985 | Japan | Furubira | 43.27 | 140.67 | 580.00 | 1700 | F | set net | scientific confirmation | Nakano and Nakaya 1987 [2] |
| April 2, 1986 | Japan | Taiji, Honshu | 33.33 | 135.46 | 470.00 |  | F† |  | scientific confirmation | Uchida et al. 1996 [1] |
| Feburary 14, 1988 | Japan | Nago Bay, Okinawa | 26.57 | 127.94 | 380.00 | 543 | M | set line | scientific confirmation | Uchida et al. 1996 [1] |
| August 7, 1989 | Japan | IE Island, Okinawa | 26.70 | 127.79 | 301.00 | 270 | M | set line | scientific confirmation | Uchida et al. 1996 [1] |
| August 15, 1989 | Japan | IE Island, Okinawa | 26.70 | 127.79 | 508.00 | 1325 | F | set line | scientific confirmation | Uchida et al. 1996 [1] |
| August 19, 1989 | Japan | IE Island, Okinawa | 26.70 | 127.79 | 435.00 | 850 | M | set line | scientific confirmation | Uchida et al. 1996 [1] |
| 1990 | Japan |  |  |  | 560.00 |  |  |  | personal communication | Nishida personal communication |
| November 2, 1990 | Japan | IE Island, Okinawa | 26.70 | 127.79 | 436.00 | 830 | F | set line | scientific confirmation | Uchida et al. 1996 [1] |
| November 2, 1990 | Japan | IE Island, Okinawa | 26.70 | 127.79 | 460.00 | 1100 | M | set line | scientific confirmation | Uchida et al. 1996 [1] |
| March 8, 1992 | Japan | Matsuyama, Ehime | 33.85 | 132.67 |  |  |  |  | scientific confirmation | Nakaya 1994 [3] |
| March 11, 1992 | Japan | Sata, Kagoshima | 30.99 | 130.65 | 370.00 | 552* | M | gill net | scientific confirmation | Nakaya 1994 [3] |
| April 18, 1992 | Japan | Ashizuri, Kochi | 32.77 | 133.02 | 375.00 | 400 | F | set net | scientific confirmation | Nakaya 1994 [3] |
| April 21, 1992 | Japan | Shimamaki, Hokkaido | 42.72 | 140.02 | 552.00 | 2500 | F | set net | scientific confirmation | Nakaya 1994 [3] |
| May 14, 1992 | Japan | Uchinoura, Kagoshima | 31.36 | 130.74 | 480.00 | 1500* | F† | set net | scientific confirmation | Nakaya 1994 [3] |
| May 22, 1992 | Japan | Toyo, Kochi | 33.51 | 134.30 | 515.00 | 2500 | F† | set net | scientific confirmation | Nakaya 1994 [3] |
| May 22, 1992 | Japan | Harima-nada, Hyogo | 34.59 | 135.25 | 490.00 | 1100 | M | trawl | scientific confirmation | Nakaya 1994 [3] |
| May 22, 1992 | Japan | Mizunoko Island, Ttsurumi-Machi, Ooita | 33.01 | 132.10 | 274.00 | 200* |  | Purse Seine | personal communication/photo | Author observation |
| May 29, 1992 | Japan | Furubira, Hokkaido | 43.27 | 140.67 | 538.00 | 2000 | F | set net | scientific confirmation | Nakaya 1994 [3] |
| June 10, 1992 | Japan | Ishinomaki, Miyagi | 38.35 | 141.30 | 300.00 | 400 | M | set net | scientific confirmation | Nakaya 1994 [3] |
| June 17, 1992 | Japan | Taisei, Ikata, Ehime | 33.41 | 132.29 | 500.00 |  |  |  | scientific confirmation | Nakaya 1994 [3] |
| July 7, 1992 | Japan | Ugui, Nachi-Katsuura, Wakayama | 33.62 | 135.99 | 450.00 | 800 | M | set net | scientific confirmation | Nakaya 1994 [3] |
| August 5, 1992 | Japan | Nobeoka City, Miyazaki | 32.57 | 131.76 | 400.00 | 1200 |  | Bottom trawl net | personal communication | Author observation |
| December 29, 1992 | Japan | Chikura, Chiba | 34.98 | 139.99 | 570.00 |  |  | set net | scientific confirmation | Nakaya 1994 [3] |
| January 1993 | Japan | Uchinoura, Kagoshima | 31.35 | 130.72 | 250.00 |  |  |  | scientific confirmation | Nakaya 1994 [3] |
| January 4, 1993 | Japan | off Namitu, Okagaki- Machi, Fukuoka | 33.90 | 130.59 | 402.00 | 460 | M | set net | scientific confirmation | Nakaya 1994 [3] |
| January 4, 1993 | Japan | Taki, Shimane | 35.24 | 132.28 | 350.00 |  | M | set net | scientific confirmation | Nakaya 1994 [3] |
| March 4, 1993 | Japan | Chikura, Chiba | 34.98 | 139.99 | 460.00 |  |  | set net | scientific confirmation | Nakaya 1994 [23 |
| March 17, 1993 | Japan | Iyo, Ehime | 33.75 | 132.70 |  |  |  |  | scientific confirmation | Nakaya 1996 [4] |
| April 15, 1993 | Japan | Narahara, Oita | 33.36 | 131.82 | 436.00 | 1300 | M | gill net | scientific confirmation | Nakaya 1994 [3] |
| August 5, 1993 | Japan | Nishinoshima, Shimane | 36.07 | 132.99 | 500.00 |  | M | set net | scientific confirmation | Nakaya 1994 [3] |
| 1994 | Japan |  |  |  | 490.00 |  |  |  | personal communication | Shimamoto personal communication |
| January 4, 1994 | Japan | Shimoda, Shizuoka | 34.64 | 138.95 | 420.00 |  | M | crab basket | scientific confirmation | Nakaya 1994 [3] |
| March 30, 1994 | Japan | Otuki-Machi, Hatata, Kouchi | 32.78 | 132.71 | 480.00 | 1500 | F† | set net | personal communication | Head and two embryos preserved in Suma Aqualife Park |
| April 9, 1995 | Japan | Atsumi-cho, Aichi | 34.50 | 137.21 | 520.00 |  |  |  | scientific confirmation | Nakaya 1996 [4] |
| April 11,1995 | Japan | Ryotsu City, Sado Island | 38.10 | 138.47 | 420.00 | 600* | M | set net | personal communication | Author observation |
| May 28, 1996 | Japan | Kamishima, Toba-City, Mie | 34.53 | 137.01 | 273.00 |  |  | Purse seine | personal communication | Author observation |
| July 3, 1996 | Japan | Tachikawa, Sumoto City, Hyougo | 34.26 | 134.99 | 150.00 | 30 |  | Bottom trawl | personal communication/photo | Photo |
| July 7, 1996 | Japan | Tubakitomari-Machi, Anan City, Tokushima | 33.91 | 134.80 | 280.00 | 200 |  |  | personal communication/photo | Photo |
| July, 1996 | Japan | Aise, Ibaraki | 36.58 | 140.66 | 400.00 | 540 | M | set net | preserved | Dry specimen in Oarai Aquarium |
| August 1, 1996 | Japan | Watari-machi, Miyagi | 38.04 | 141.05 | 300.00 | 250* |  | bottom gill net | personal communication | Author observation |
| July 23, 1996 | Japan | Hirara City, Miyako, Okinawa | 24.77 | 125.23 | 250.00 |  |  |  | media report | M. Shimbun; japanupdate.com |
| May 18, 1997 | Japan | Shiura, Minamijima-machi, Mie | 34.25 | 136.57 | 500.00 | 1300 | F† | set net | personal communication | Author observation |
| July 12, 1997 | Japan | Hirara City, Miyako, Okinawa | 24.77 | 125.23 |  |  |  |  | media report | Okinawa Weekly Times, 7/19/1997 editon; Ryukyu Shrimpo News (Japan) |
| December 12, 1998 | Japan | Urasiro-machi, Nobeoka, Miyazaki | 32.65 | 131.81 | 400.00 |  |  | Set net | personal communication | Author observation |
| July 8, 1999 | Japan | Hikari City, Yamaguchi | 33.94 | 131.92 | 520* | 3000* | F |  | preserved | Jaws preserved in Kaikyokan Aquarium |
| July 5, 2000 | Japan | Taiji, Wakayama | 33.60 | 135.98 | 300.00 | 250 | M | Harpoon | personal communication | Author observation |
| September 16, 2000 | Japan | Sunayama Beach, Miyako, Okinawa | 24.82 | 125.37 | 250* |  |  | attack | media report | http://sharkattacksurvivors.com/shark_attack/viewtopic.php?f=7&t=304&p=662&hilit=japan#p662 |
| December 13, 2000 | Japan | Aise, Ibaraki | 36.58 | 140.66 | 400.00 |  | M | set net | personal communication | Author observation |
| May, 2001 | Japan | off Owase, Mie | 34.07 | 136.26 | 400.00 | 300 |  | set net | photo | photo |
| April 14, 2003 | Japan | Kashima-nada off Aise, Ibaraki | 36.58 | 140.66 | 315.00 |  |  | set net | scientific confirmation | Measurement by Ibaraki Prefectural Oarai Aquarium staff |
| June 22, 2003 | Japan | Kashima-nada off Aise, Ibaraki | 36.58 | 140.66 | 350.00 |  |  | set net | scientific confirmation | Measurement by Ibaraki Prefectural Oarai Aquarium staff |
| April 30, 2004 | Japan | Kashima-nada off Aise, Ibaraki | 36.58 | 140.66 | 360.00 | 515 | F | set net | scientific confirmation | Measurement by Ibaraki Prefectural Oarai Aquarium staff |
| May 16, 2004 | Japan | Kashima-nada off Aise, Ibaraki | 36.58 | 140.66 | 320.00 | 356 | F | set net | scientific confirmation | Measurement by Ibaraki Prefectural Oarai Aquarium staff |
| June 28, 2004 | Japan | Kashima-nada off Aise, Ibaraki | 36.58 | 140.66 | 411.00 | 572 | M | set net | scientific confirmation | Measurement by Ibaraki Prefectural Oarai Aquarium staff |
| November 19, 2004 | Japan | Nobeoka, Miyazaki | 32.65 | 131.81 | 250.00 | 170 | M | set net | scientific confirmation | Kept one day in Ooita aquarium |
| April 8, 2005 | Japan | Kashima-nada off Aise, Ibaraki | 36.58 | 140.66 | 381.00 | 684 | M | set net | scientific confirmation | Measurement by Ibaraki Prefectural Oarai Aquarium staff |
| April 14, 2005 | Japan | Kashima-nada off Aise, Ibaraki | 36.58 | 140.66 | 406.00 | 663 | M | set net | scientific confirmation | Measurement by Ibaraki Prefectural Oarai Aquarium staff |
| April 20, 2005 | Japan | Kashima-nada off Aise, Ibaraki | 36.58 | 140.66 | 442.00 | 828 | M | set net | scientific confirmation | Measurement by Ibaraki Prefectural Oarai Aquarium staff |
| April 22, 2005 | Japan | Kashima-nada off Aise, Ibaraki | 36.58 | 140.66 | 398.00 | 861 | F | set net | scientific confirmation | Measurement by Ibaraki Prefectural Oarai Aquarium staff |
| April 24, 2005 | Japan | Kashima-nada off Aise, Ibaraki | 36.58 | 140.66 | 482.00 | 1138 | F | set net | scientific confirmation | Measurement by Ibaraki Prefectural Oarai Aquarium staff |
| May 18, 2005 | Japan | Kashima-nada off Aise, Ibaraki | 36.58 | 140.66 | 353.00 | 525.4 | M | set net | scientific confirmation | Measurement by Ibaraki Prefectural Oarai Aquarium staff |
| May 27, 2005 | Japan | Kashima-nada off Aise, Ibaraki | 36.58 | 140.66 | 324.00 | 342 | M | set net | scientific confirmation | Measurement by Ibaraki Prefectural Oarai Aquarium staff |
| June 2, 2005 | Japan | Kashima-nada off Aise, Ibaraki | 36.58 | 140.66 | 383.00 | 518.5 | F | set net | scientific confirmation | Measurement by Ibaraki Prefectural Oarai Aquarium staff |
| June 8, 2005 | Japan | Kashima-nada off Aise, Ibaraki | 36.58 | 140.66 | 426.00 | 748 | M | set net | scientific confirmation | Measurement by Ibaraki Prefectural Oarai Aquarium staff |
| June 14, 2005 | Japan | Kashima-nada off Aise, Ibaraki | 36.58 | 140.66 | 232.00 | 138.2 | F | set net | scientific confirmation | Measurement by Ibaraki Prefectural Oarai Aquarium staff |
| October 26, 2005 | Japan | Chidori Canal, Yako, Kawasaki | 35.48 | 139.77 | 481.00 | 1110 | M |  | photo | Author observation |
| April 24, 2006 | Japan | off Houzaura, Minami-Isemachi, Mie | 34.24 | 136.52 | 320.00 | >250 |  | set net | personal communication | Author observation |
| 2007 | Japan | Kōchi County | 33.45 | 133.54 | 530.00 |  | F | set net | personal communication | Author observation |
| 2007 | Japan |  |  |  | 420.00 | 550 |  |  | photo | Photos of jaws posted on blog |
| 2007 | Japan |  |  |  | 480* |  |  |  | preserved | Jaws on auction |
| January 18, 2007 | Japan | Okinawa | 26.04 | 127.54 | 480.00 | 1300 | F† |  | personal communication | Author observation |
| January 31, 2007 | Japan | Kesennuma fish market | 38.80 | 141.62 | 430.00 | 700 | M |  | personal communication | Author observation |
| May 15, 2007 | Japan | Kagoshima | 31.53 | 130.57 | 480.00 |  |  | set net | preserved | Jaws preserved in unknown museum |
| 2008 | Japan |  |  |  | 500.00 | 1000 |  |  | photo | Photos of jaws posted on blog |
| 2008 | Japan | Iwate | 39.69 | 142.13 |  | 500* |  |  | personal communication | Author observation |
| February 24, 2008 | Japan | Kashima-nada off Aise, Ibaraki | 36.58 | 140.66 | 230.00 | 138.5 | M | set net | scientific confirmation | Measurement by Ibaraki Prefectural Oarai Aquarium staff |
| March 1, 2008 | Japan | Kashima-nada off Aise, Ibaraki | 36.58 | 140.66 | 365.00 | 422.0 | M | set net | scientific confirmation | Measurement by Ibaraki Prefectural Oarai Aquarium staff |
| March 3, 2008 | Japan | Kashima-nada off Aise, Ibaraki | 36.58 | 140.66 | 231.00 | 124.2 | M | set net | scientific confirmation | Measurement by Ibaraki Prefectural Oarai Aquarium staff |
| April 14, 2008 | Japan | Kashima-nada off Aise, Ibaraki | 36.58 | 140.66 | 280.00 | 234.0 | Ｆ | set net | scientific confirmation | Measurement by Ibaraki Prefectural Oarai Aquarium staff |
| March 27, 2008 | Japan | Kashima-nada off Aise, Ibaraki | 36.58 | 140.66 | 446.00 | 970.0 | Ｆ | set net | scientific confirmation | Measurement by Ibaraki Prefectural Oarai Aquarium staff |
| January 22, 2009 | Japan | Iwate | 39.64 | 142.14 |  | 400 |  | set net | personal communication | Author observation |
| April 20, 2009 | Japan | Kashima-nada off Aise, Ibaraki | 36.58 | 140.66 | 411.00 | 889.0 | M | set net | scientific confirmation | Measurement by Ibaraki Prefectural Oarai Aquarium staff |
| April 24, 2009 | Japan | Kashima-nada off Aise, Ibaraki | 36.58 | 140.66 | 311.00 | 320.0 | M | set net | scientific confirmation | Measurement by Ibaraki Prefectural Oarai Aquarium staff |
| June 10, 2009 | Japan | Rikuzen-Takada, Iwate | 38.94 | 141.69 | 300.00 | 350 | F | set net | personal communication | Author observation |
| December 27, 2009 | Japan | Kashima-nada off Aise, Ibaraki | 36.58 | 140.66 | 408.00 | 717.5 | M | set net | scientific confirmation | Measurement by Ibaraki Prefectural Oarai Aquarium staff |
| January 9, 2010 | Japan | Aomori fish market | 40.94 | 140.74 |  | 800(550 gutted) | M |  | personal communication | Author observation |
| March 18, 2010 | Japan |  |  |  | 300.00 |  | F |  | personal communication | Author observation |
| January 28, 2011 | Japan | off Heiizaki, Miyako | 39.66 | 142.03 | 440* | 640 | M | set net | personal communication | Author observation |
| February 17, 2011 | Japan | Ise Bay, near Kozukumi Island off Toba City, Mie | 34.55 | 136.92 | 340.00 |  |  |  | personal communication | Author observation |
| April 26, 2011 | Japan |  |  |  | 400* |  |  |  | personal communication | Author observation |
| January 27, 2012 | Japan | Kashima-nada off Aise, Ibaraki | 36.58 | 140.66 | 360.00 |  | M | set net | scientific confirmation | Measurement by Ibaraki Prefectural Oarai Aquarium staff |
| February 1, 2012 | Japan | Kashima-nada off Aise, Ibaraki | 36.58 | 140.66 | 342.00 | 480 | F | set net | scientific confirmation | Measurement by Ibaraki Prefectural Oarai Aquarium staff |
| February 1, 2012 | Japan | Kagoshima fish market | 31.52 | 130.63 | 250* |  |  |  | photo | Author observation |
| May 1, 2003 | Philippines | Limay, Baatan | 14.52 | 120.63 |  |  |  |  | media report | D. Cervantes, Star http://www.philstar.com/nation/207333/man-eating-white-sharks-sighted-bataan |
| June 9, 1951 | Russia | Tatar Strait, Sakhalin | 47.32 | 141.43 | 477 | 894 |  |  | scientific confirmation | Dolganov 2012 [5], Velikanov 2010[6], Probatov 1952 [7] |
| 1984 | Russia | Cape Gamov |  |  | 200 |  |  |  | scientific confirmation | Dolganov 2012 [5] |
| 1999 | Russia | Primoriye, Olga Bay | 43.69 | 135.25 | 400 |  |  |  | personal communication | Author observation |
| July 30, 2004 | Russia | Kunahir Island | 44.49 | 146.84 |  |  |  |  | media report | M. Gozum & J. Eager, scubaradio.com |
| July 19, 2007 | Russia | Aniva Bay, Sakhalin | 46.41 | 142.61 | 504.00 | 1111 | M | seine | scientific confirmation | Velikanov 2010 [6] |
| September 25, 2011 | Russia | Primorye, Popov Island |  |  | 126.00 | 16 |  |  | scientific confirmation | Dolganov 2012 [5] |
| August 17, 2011 | Russia | Primosky Krai | 44.57 | 136.64 |  |  |  |  | scientific confirmation/ media | http://www.nytimes.com/2011/08/19/world/europe/19jaws.html |
| August 18, 2011 | Russia | Zheltukhina Island | 42.82 | 131.61 |  |  |  |  | media report | http://www.huffingtonpost.com/2011/08/19/two-injured-in-unpreceden_n_931156.html |
|  | Republic of Korea |  |  |  | 350* |  |  |  | personal communication | Author observation |
| Summer 1959 | Republic of Korea |  | 37.28 | 126.52 |  |  |  |  | personal communication | Y. Choi & K. Nakaya |
| May 23, 1981 | Republic of Korea | Wae-yeon Island Chungman | 36.35 | 126.19 | 600.00 |  |  |  | personal communication | Y. Choi & K. Nakaya |
| June 1996 | Republic of Korea |  |  |  | 480.00 |  | M |  | preserved | Mounted at Busan Marine Natural History Museum |
| May 1, 1998 | Republic of Korea |  |  |  | 500* |  |  |  | personal communication | Author observation |
| April 14, 2000 | Republic of Korea |  |  |  |  |  |  |  | personal communication | Author observation |
| April 25, 2005 | Republic of Korea |  |  |  |  | 1500 | F |  | personal communication | Author observation |
| June 14, 2005 | Republic of Korea | Kaeui Island, South Chungchong | 36.42 | 126.15 | 300.00 |  |  |  | media report | http://sharkattackmonitor.wordpress.com/2005/06/22/injured-14-june-2005-kaeui-island-korea-diver-attacked/ |
| April 4, 2007 | Republic of Korea |  |  |  | 460.00 |  |  |  | personal communication | Author observation |
| February 1, 2009 | Republic of Korea | Mukho | 37.52 | 129.27 | 350.00 | 2000 |  |  | media report | http://www.koreatimes.co.kr/www/news/nation/2009/07/117_47190.html |
| March 1, 2009 | Republic of Korea | Mukho | 37.52 | 129.27 | 470.00 | 1360* |  |  | media report | http://www.koreatimes.co.kr/www/news/nation/2009/07/117_47190.html |
| March 6, 2009 | Republic of Korea |  |  |  | 500* |  |  |  | photo | Photos of being dissembled shown on internet |
| March 9, 2009 | Republic of Korea |  |  |  | 440.00 |  |  |  | personal communication | Author observation |
| March 27, 2009 | Republic of Korea | Mukhohang | 37.57 | 129.21 | 440* | 1500 | F |  | personal communication | Author observation |
| August 10, 2009 | Republic of Korea | Eojongin |  |  | 545.00 | 1000 | F |  | personal communication | Author observation |
| August 10, 2009 | Republic of Korea | Incheon | 37.38 | 126.30 | 470.00 | 800 |  |  | personal communication | Author observation |
| 2010 | Republic of Korea |  |  |  |  |  |  |  | photo | Photos of being dissembled shown on internet |
| 2010 | Republic of Korea |  |  |  | 130.00 |  |  |  | personal communication | Author observation |
| 2010 | Republic of Korea |  |  |  | 360* |  |  |  | photo | Author observation |
| 2010 | Republic of Korea |  |  |  | 400* |  |  |  | photo | Author observation |
| 2010 | Republic of Korea |  |  |  | 500* | 1200* |  |  | photo | Photos of being dissembled shown on internet |
| 2011 | Republic of Korea | Donghae | 37.53 | 129.16 | 560* | 2000* |  |  | photo | TL and mass estimated from the photo |
| ~1980 | Taiwan |  |  |  |  | 2100 |  |  | personal communication | Li personal communication. Teeth preserved. |
| 1983 | Taiwan |  |  |  | 400* |  |  |  | preserved | Captured by Taiwanese fishing boat. Jaws shown on Japanese website |
| Feb-March 1988 | Taiwan | Keelung | 25.18 | 121.74 |  | 3000* | F† |  | personal communication | Dave Ebert personal communication |
| 1990 | Taiwan | ChenKung, Taitung | 22.72 | 121.16 |  | 700 |  |  | preserved | Jaws preserved in local museum |
| 1990 | Taiwan | Southern Bay | 21.94 | 120.77 |  | 809 |  |  | personal communication | Chang personal communication |
| 1990 | Taiwan | Southern Bay | 21.94 | 120.77 |  | 1000 |  |  | personal communication | Zhuang personal communication |
| 1990 | Taiwan | Southern Bay | 21.94 | 120.77 |  | 1000 | M |  | personal communication | Huang personal communication |
| 1990 | Taiwan | ChenKung, Taitung | 22.72 | 121.16 |  | 1100 |  |  | preserved | Jaws preserved in local museum |
| 1990 | Taiwan | Southern Bay | 21.94 | 120.77 |  | 1200* |  |  | personal communication | Chen personal communication |
| 1990 | Taiwan | Southern Bay | 21.94 | 120.77 |  | 1200 |  |  | personal communication | Hsu personal communication |
| 1990 | Taiwan | ChenKung, Taitung | 22.72 | 121.16 |  | 1300 | F |  | preserved | Jaws preserved in local museum |
| 1990 | Taiwan | Southern Bay | 21.94 | 120.77 |  | 2050* |  |  | personal communication | Author observation |
| 1995 | Taiwan |  |  |  | 400 * |  |  |  | preserved | Teeth preserved |
| 1995 | Taiwan |  |  |  | 400* | 800 |  |  | preserved | Jaws preserved for sale |
| October 1995 | Taiwan | ChenKung, Taitung | 22.72 | 121.16 | 500* | 1400 | F |  | preserved | Whole specimen in local museum |
| May 1997 | Taiwan | Seven Star Lake | 23.67 | 121.61 | 670* | 2500 | F†† |  | preserved | Jaws preserved in local museum. |
| October 13, 1997 | Taiwan | Baisolian, Taitung | 22.72 | 121.16 |  | 2000 | F† |  | preserved | Jaws, fins, vertebrae and embryos preserved |
| 1999 | Taiwan | ChenKung, Taitung | 22.72 | 121.16 |  | 1500 | F |  | preserved | Jaws preserved in local museum |
| October 17, 2000 | Taiwan | Southern Bay | 21.94 | 120.77 | 340.00 | 367 | F |  | preserved | Jaws preserved |
| October 23, 2000 | Taiwan | SanShenTai, Taitung | 22.72 | 121.16 | 500* | 1200 | M |  | personal communication | Author observation |
| 2002 | Taiwan | Southern Bay | 21.94 | 120.77 |  | 720 | F |  | personal communication | Author observation |
| 2003 | Taiwan | Seven Star Lake | 23.67 | 121.61 |  | 810* |  |  | preserved | Jaws preserved, Shou personal communication |
| 2003 | Taiwan | Southern Bay | 21.94 | 120.77 |  | 130 |  |  | personal communication | Author observation |
| January 2, 2004 | Taiwan | S.E. Taiwan | 22.36 | 121.13 | 557* | 2120 |  |  | preserved | Vertebrae preserved |
| December 17, 2004 | Taiwan | ChenKung, Taitung | 22.72 | 121.16 | 500* | 1400* |  |  | photo | Brought to ChenKung fish market |
| 2005 | Taiwan | Southern Bay | 21.94 | 120.77 |  | 400 |  |  | personal communication | Author observation |
| October 13, 2005 | Taiwan | Seven Star Lake | 23.67 | 121.61 | 531.00 | 1730* | F |  | media report | Author observation |
| 2005 | Taiwan | Kauhsaung | 22.65 | 120.16 |  | 2000 |  |  | preserved | Jaws preserved. |
| 2006 | Taiwan | Southern Bay | 21.94 | 120.77 |  | 53 | F |  | personal communication | Author observation |
| 2006 | Taiwan | Seven Star Lake | 23.67 | 121.61 |  | 425 |  |  | personal communication | Author observation |
| 2006 | Taiwan | Seven Star Lake | 23.67 | 121.61 | 400* | 590 | F |  | personal communication | Author observation |
| 2007 | Taiwan |  |  |  |  | 800 |  |  | preserved | Jaws put on auction |
| 2008 | Taiwan | DaLi, I-Lan | 24.96 | 121.92 |  | 800 | M |  | personal communication | Author observation |
| 2008 | Taiwan | Southern Bay | 21.94 | 120.77 |  | 107 |  |  | personal communication | Author observation |
| November 7, 2008 | Taiwan | brought to Southern Bay Dock, East Taiwan | 23.57 | 121.91 | 542.00 | 1930 | F† |  | personal communication | Author observation |
| February 17, 2009 | Taiwan | Taitung | 22.72 | 121.16 | 500* | 1020 | M |  | personal communication | Author observation |
| 2009 | Taiwan |  |  |  |  | 209 |  |  | preserved | Author observation |
| 2009 | Taiwan | DaShi, I-Lan | 24.89 | 121.16 |  | 130 |  |  | preserved | Jaws preserved |
| October 20, 2009 | Taiwan | Baisolian, Taitung | 22.72 | 121.16 | 533.00 | 1800* | F |  | photo | Author observation |
| 2010 | Taiwan | Southern Bay | 21.94 | 120.77 |  | 700* |  |  | personal communication | Wang personal communication |
| 2010 | Taiwan | ChenKung, Taitung | 22.72 | 121.16 |  | 800* |  |  | personal communication | Author observation |
| October 27, 2012 | Taiwan | Northeast |  |  | 500* | 1500* | F | set net | Personal communication | Author observation |
| November 2, 2012 | Taiwan |  |  |  | 500.00 | 1286 | F |  | personal communication | Author observation |
| November 2, 2012 | Taiwan |  |  |  | 450.00 | 900 | F† |  | personal communicaiton | Author observation |
| June 14, 2009 | Vietnam |  |  |  |  |  |  |  | media report | Tin Tuc online, 6/14/2009 |
| February 4, 2010 | Vietnam | Quy Nhon City | 13.12 | 109.69 | 500.00 | 1000 |  |  | personal communication | Luis Kmentt and Helmut Nickel personal communication |
| June 1, 2011 | Vietnam | Qui Nhon Bay Binh Dinh |  |  | 1.60 | 60 |  | set net | media report | http://travelweekly.asia/news/tourism/3689-shark-scare-at-vietnam-resort |
|  |  |  |  |  |  |  |  |  |  |  |

* Estimated length or weight based on photo or calculated based on jaw measurements

† Pregnant female

†† Suspected pregnant female

**References**

1. Uchida S, Todo M, Teshima K, Yano K (1996) Pregnant white sharks and full-term embryos from Japan. In: Klimley AP, Ainley DG, editors. Great White Sharks: The Biology of *Carcharodon carcharias*. San Diego: Academic Press. pp. 139-155.

2. Nakano H, Nakaya K (1987) Records of the white shark *Carcharodon carcharias* from Hokkaido, Japan. Jpn J Ichthyol 33: 414-416.

3. Nakaya K (1994) Distribution of white shark in Japanese waters. Fish Sci 60: 515-518.

4. Nakaya K (1996) A fatal shark attack in Aichi Prefecture, Japan, with other confirmed attack cases in Japanese water. Fish Sci 62: 830-831.

5. Dolganov VN (2012) The capture of a great white shark *Carcharodon carcharias* Linnaeus, 1758 (Carcharodontidae) in Peter the Great Bay (Sea of Japan). Russ J Mar Biol 38: 88-90.

6. Velikanov AY (2010) The record of the white shark *Carcharodon carcharias* (Lamnidae) from Aniva Bay, Sakhalin. J Ichthyol 50: 347-350.

7. Probatov AN (1952) A Shark at the Coast of Sakhalin, Priroda 6: 115.
