# Supplementary material for: The Last Frontier: Catch Records of White Sharks (Carcharodon carcharias) in the Northwest Pacific Ocean
Source: PLoS One. 2014 Apr 16;9(4):e94407. doi: 10.1371/journal.pone.0094407 (PMC3989224; doi:10.1371/journal.pone.0094407)
Supplement: Table S2 — Global records of white shark embryos and free-swimming juveniles. (DOC) [file pone.0094407.s003.doc]

**Table S2- Global records of white shark embryos and free-swimming juveniles**

| **Date of Capture** | **Location** | **Total Length (cm)** | **Stage** | **Source** |
| --- | --- | --- | --- | --- |
| August 1, 1976 | Rhode Island, USA | 122 | Embryo | R. Ellis pc |
| July 16, 1996 | Malindi Kenya | 110 | Embryo | Cliff et al. 2000 [1] |
| Summer 1934 | alexandria, egypt | 61 | Embryo | Norman & Fraser 1937 [2], Ellis and McCosker 1991 [3] |
| February 26, 2004 | Gulf of Gabes, southern Tunisia | 132 | Embryo | Saïdi et al. 2005 [4] |
| September 1936 | California, USA | 167.6 | Free-swimming | Cailliet et al. 1985 [5] |
| April 18, 1974 | North Carolina, USA | 197 | Free-swimming | G. Burgess p.c. |
| August 1975 | Rhode Island, USA | 127 | Free-swimming | Ellis and McCosker 1991 [3] |
| July 1976 | California, USA | 136 | Free-swimming | Cailliet et al. 1985 [5] |
| July 1976 | California, USA | 150 | Free-swimming | Cailliet et al. 1985 [5] |
| October 1979 | California, USA | 183 | Free-swimming | Cailliet et al. 1985 [5] |
| August 1980 | California, USA | 146 | Free-swimming | Cailliet et al. 1985 [5] |
| June 1981 | California, USA | 129 | Free-swimming | Cailliet et al. 1985 [5] |
| August 1981 | California, USA | 147 | Free-swimming | Cailliet et al. 1985 [5] |
| August 3, 1981 | California, USA | 160 | Free-swimming | Ellis and McCosker 1991 [3] |
| September 1981 | California, USA | 159 | Free-swimming | Cailliet et al. 1985 [5] |
| August 1983 | California, USA | 167 | Free-swimming | Cailliet et al. 1985 [5] |
| July 1, 1984 | California, USA | 150 | Free-swimming | Hewitt 1984 [6] |
| September 10, 1984 | California, USA | 154 | Free-swimming | Mollet et al. 1996 [7] |
| September 10, 1984 | California, USA | 154 | Free-swimming | Ellis and McCosker 1991 [2] |
| June 18, 1985 | California, USA | 126 | Free-swimming | J. Seigel p.c. |
| January 6, 1994 | Georgia, USA | 183 | Free-swimming | G. Burgess p.c. |
| May 30, 2000 | California, USA | 140 | Free-swimming | Dewar et al. 2004 [8] |
| August 20, 2004 | California, USA | 141 | Free-swimming | Ezcurra et al. 2012 [9] |
| August 17, 2006 | California, USA | 174 | Free-swimming | Ezcurra et al. 2012 [9] |
| August 4, 2007 | California, USA | 143 | Free-swimming | Ezcurra et al. 2012 [9] |
| August 16, 2008 | California, USA | 137 | Free-swimming | Ezcurra et al. 2012 [9] |
| August 13, 2009 | California, USA | 157 | Free-swimming | Ezcurra et al. 2012 [9] |
| November 13, 1991 | North Cape, New Zealand | 143 | Embryo | Francis 1996 [10] |
| November 9, 2003 | Waiheke Island, New Zealand | 150 | Embryo | New Zealand Herald |
| October or November | West Coast Australia | 30 | Embryo | Bruce 1992 [11] |
| October or November | West Coast Australia | 5 | Embryo | Bruce 1992 [11] |
| March 1, 1994 | South Australia | 127 | Embryo | JD Stevens p.c. |
| September 27, 1964 | South Africa | 170 | Free-swimming | Francis 1996 [10] |
| March 1, 1967 | NSW Australia | 139 | Free-swimming | Francis 1996 [10] |
| March 1, 1981 | NSW Australia | 153 | Free-swimming | Francis 1996 [10] |
| December 1, 1984 | NSW Australia | 146 | Free-swimming | Francis 1996 [10] |
| January 1, 1985 | South Africa | 159 | Free-swimming | Francis 1996 [10] |
| May 1, 1986 | South Africa | 151 | Free-swimming | Francis 1996 [10] |
| December 15, 1989 | Eastern Cape, South Africa | 160 | Free-swimming | Francis 1996 [10] |
| December 15, 1989 | South Africa | 160 | Free-swimming | Francis 1996 [10] |
| September 14, 1991 | New Zealand | 155 | Free-swimming | Francis 1996 [10] |
| January 15, 1992 | New Zealand | 174 | Free-swimming | Francis 1996 [10] |
| January 18, 1992 | NSW Australia | 147 | Free-swimming | Francis 1996 [10] |
| January 19, 1993 | New Zealand | 152 | Free-swimming | Francis 1996 [10] |
| March 1, 2000 | Australia | 180 | Free-swimming | Bruce and Bradford 2012 [12] |
| October 8, 2007 | Australia | 190 | Free-swimming | Bruce and Bradford 2012 [12] |
| October 11, 2007 | Australia | 190 | Free-swimming | Bruce and Bradford 2012 [12] |
| October 28, 2008 | Australia | 175 | Free-swimming | Bruce and Bradford 2012 [12] |

p.c. –personal communication

**References**

1. Cliff G, Compagno LJV, Smale MJ, van der Elst RP, Wintner SP (2000) First records of white sharks, *Carcharodon carcharias*, from Mauritius, Zanzibar, Madagascar and Kenya. S Afr J Sci 96: 365-367.

2. Norman JR, Fraser FC (1937) Giant Fishes, Whales and Dolphins. Putnam, London. 376 p.

3. Ellis R, McCosker JE (1991) Great white shark. Stanford: Standford University Press. 270 p.

4. Saïdi B, Bradaï MN, Bouan A, Guélorget O, Capapé C (2005) Capture of a pregnant female white shark, *Carcharodon carcharias* (Lamnidae) in the Gulf of Gabès (southern Tunisia, central Mediterranean) with comments on oophagy in sharks. Cybium 29: 303-307.

5. Cailliet GM, Natanson LJ, Welden BA, Ebert DA (1985) Preliminary studies on the age and growth of the white shark, *Carcharodon carcharias,* using vertebral bands. Mem South Calif Acad Sci 9: 49–60.

6. Hewitt JC (1984) The great white shark in captivity: a history and prognosis. AAZPA Annual Proceedings. pp. 317-324.

7. Mollet HF, Cailliet GM, Klimley AP, Ebert DA, Testi AD, et al. (1996) A review of length validation methods and protocols to measure large white sharks. In: Klimley AP, Ainley DG, editors. Great White Sharks: The Biology of *Carcharodon carcharias*. San Diego: Academic Press. pp. 91-108.

8. Dewar H, Domeier M, Nasby-Lucas N (2004) Insights into young of the year white shark, *Carcharodon carcharias,* behavior in the Southern California Bight. Environ Biol Fish 70: 133-143.

9. Ezcurra JM, Lowe CG, Mollet HF, Ferry LA, O'Sullivan JB (2012) Captive feeding and growth of young-of-the-year white sharks, *Carcharodon carcharias*, at the Monterey Bay Aquarium. In: Domeier ML, editor. Global Perspectives on the Biology and Life History of the White Shark. Boca Raton: CRC Press. pp. 3-15.

10. Francis MP (1996) Observations on a pregnant white shark with a review of reproductive biology. In: Klimley AP, Ainley DG, editors. Great White Sharks: The Biology of *Carcharodon carcharias*. San Diego: Academic Press. pp. 157-172.

11. Bruce BD (1992) Preliminary observations on the biology of the white shark, *Carcharodon carcharias*, in South Australian waters. Aust J Mar Fresh Res 43: 1-11.

12. Bruce BD, Bradford RW (2012) Habitat use and spatial dynamics of juvenile white sharks, *Carcharodon carcharias*, in Eastern Australia. In: Domeier ML, editor. Global Perspectives on the Biology and Life History of the White Shark. Boca Raton: CRC Press Boca Raton. pp. 225-253.
